# Pan-cancer analysis of tissue-plasma genomic concordance reveals enhanced concordance with liver metastasis and plasma clonality as predictor of poorer overall survival

**DOI:** 10.1038/s41698-026-01549-0

**Published:** 2026-07-03

**Authors:** Meng Zhang, Yi Feng, Xue Du, Changda Qu, Meizhu Meng, Meiying Ye, Min Liang, Ziran Yang, Wenjuan Gong, Xingyu Ma, Jialiang Guo, Wenmei Li, Sha Wang, Hua Bao, Shuqin Jia

**Affiliations:** 1 Key laboratory of Carcinogenesis and Translational Research (Ministry of Education/Beijing), Center for Molecular Diagnostics, Peking University Cancer Hospital & Institute, Beijing, China; 2 Geneseeq Research Institute, Nanjing Geneseeq Technology Inc, Nanjing, Jiangsu China; 3 State Key Laboratory of Holistic Integrative Management of Gastrointestinal Cancers, Center for Molecular Diagnostics, Peking University Cancer Hospital & Institute, Beijing, China

**Keywords:** Cancer genomics, Prognostic markers, Tumour biomarkers

## Abstract

Circulating tumor DNA (ctDNA) enables noninvasive tumor genotyping, yet its concordance with tissue remains unclear. Using a 437-gene panel in 1111 pan-cancer patients, we compared somatic variants between ctDNA and matched tissues. ctDNA detection sensitivity (61.5%) correlated with advanced stage (*r* = 0.955, *p* = 0.045) and tumor size (*r* = 0.955, *p* = 0.045). Actionable alterations were detected in 49.2% (ctDNA) and 77.1% (tissue) of patients. Both shared frequently mutated genes (*TP53*, APC, *KRAS*, *LRP1B*, *PIK3CA*) and pathways (RTK-RAS, p53, DNA repair). ctDNA-specific mutations were predominantly subclonal (61.5% vs. 9.7% in tissue-concordant variants) and less frequently drivers. Machine learning linked elevated concordance to progressive disease, liver metastasis, and larger tumors. ctDNA-positivity predicted worse prognosis (HR = 2.019, *p* < 0.001), exacerbated by subclonal enrichment. These findings underscore ctDNA’s capacity to reveal subclonality for risk stratification. While tissue remains superior for initial detection, ctDNA complements biopsies by capturing clonal heterogeneity.

## Introduction

Circulating tumor DNA (ctDNA) has emerged as a critical biomarker in oncology, providing a non-invasive method to interrogate tumor genomic alterations through liquid biopsy^1,2^. By detecting tumor-derived genetic variants in plasma, ctDNA facilitates real-time monitoring of therapeutic response, minimal residual disease (MRD) detection, and dynamic tracking of clonal evolution during treatment resistance^3–6^. Despite its clinical potential, the integration of ctDNA analysis into routine practice remains constrained by unresolved biological and technical limitations. A key challenge lies in the incomplete understanding of discordance between ctDNA profiles and matched tissue biopsies, which undermines confidence in liquid biopsy results and complicates clinical interpretation^7–9^.

Existing studies have identified multiple contributors to ctDNA-tissue genomic discrepancies, including spatial tumor heterogeneity, temporal clonal dynamics, and technical variability in sequencing platforms^4,10^. While prior comparisons have typically focused on single cancer types or small cohorts, the field lacks a systematic evaluation of how clinical parameters — such as metastatic organs and disease progression — influence ctDNA detectability and variant concordance^8,9,11^. Furthermore, the biological significance of ctDNA-specific mutations remains inadequately characterized, particularly regarding their clonal architecture and prognostic implications. Resolving these knowledge gaps is essential to establish ctDNA as a reliable surrogate for tissue genotyping and to optimize its clinical utility across diverse malignancies.

In this retrospective study of 1111 patients encompassing nine major cancer types, we utilize a 437-gene panel and machine learning to systematically assess ctDNA-tissue concordance. By integrating multidimensional clinical data with mutational clonality analysis, we aim to elucidate the biological significance of discordant variants in ctDNA and their implications for precision oncology.

## Results

### Demographics and clinical characteristics

The study analyzed paired blood and tissue samples from 1111 patients with nine solid tumor types, including NSCLC (*n* = 164), UGI (*n* = 223), CC (*n* = 243), RC (*n* = 181), MM (*n* = 63), BC (*n* = 95), OC (*n* = 50), SAR (*n* = 55), and PC (*n* = 37). The cohort comprised 54.9% males (610/1111) with a median age of 59 years (mean: 58.3 years, IQR: 51–67 years). Most patients presented with advanced-stage disease (stage IV: 55.2%; stages I–III: 5.4%, 8.5%, and 31.0%, respectively). Tumor diameters were ≤1 cm in 37.3% of cases, 1–2 cm in 27.2%, 2–5 cm in 24.1%, and >5 cm in 11.4%. All clinical characteristics are summarized in Supplementary Table 1. The median interval between tissue biopsy and blood sampling was 22 days (IQR: 10–92 days; Supplementary Fig. 1).

### The Concentration of cfDNA, Mean Variant Allele Frequency (mean-VAF) of ctDNA, and ctDNA Detection

**Fig. 1:**
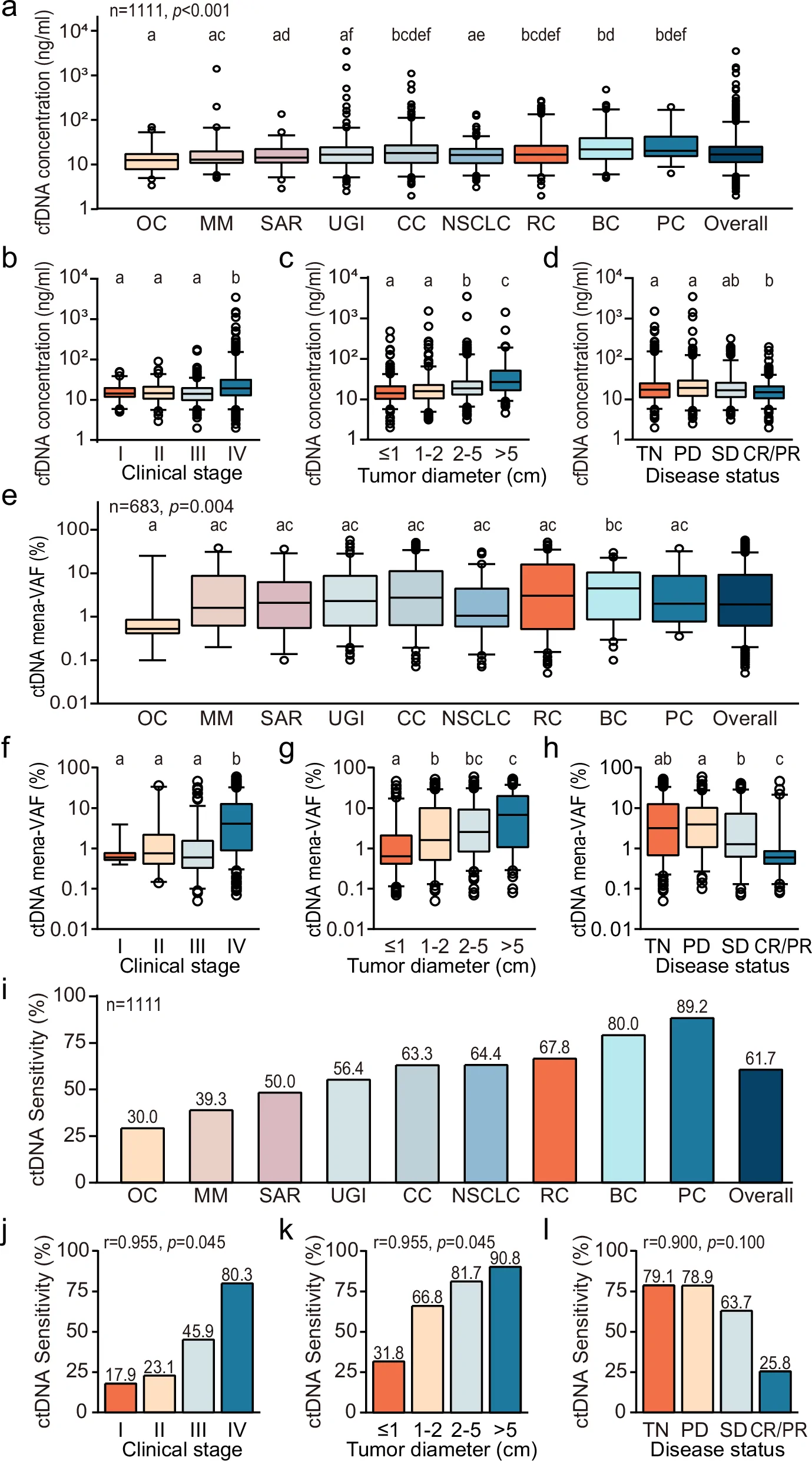
The concentration of cfDNA, mean variant allele frequency of mutations in ctDNA, and sensitivity of ctDNA. The concentration of cfDNA extracted from plasma, mean variant allele frequency of mutations in ctDNA, and detection sensitivity of ctDNA varied across different types of cancer (**a**, **e**, **i**), clinical stages (**b**, **f**, **j**), tumor diameters (**c**, **g**, **k**), and disease statuses (**d**, **h**, **l**). Letters above boxplots denote statistically homogeneous subsets determined by post hoc Dunn’s testing. Groups sharing at least one common letter indicate no significant pairwise difference. Centre line within boxes, median; box limits, upper and lower quartiles; whiskers, 5–95% percentile; rings, outliers. NSCLC non-small cell lung cancer, UGI upper gastrointestinal cancer, CC, colon cancer, RC rectal cancer, MM melanoma, BC breast cancer, OC ovarian cancer, SAR sarcoma, PC pancreatic cancer, TN treatment naïve, PD progressive disease, SD stable disease, CR complete response, PR partial response.

Plasma cfDNA concentrations varied significantly across cancer types (median: 16.8 ng/mL; range: 2.0–3514.0 ng/mL; *p* < 0.001), with higher levels observed in BC, PC, CC, and RC (Fig. 1a). Elevated cfDNA levels correlated with advanced clinical stage (Fig. 1b), larger tumor diameter (>5 cm; Fig. 1c), and progressive disease (*p* < 0.001), whereas CR/PR associated with reduced cfDNA concentrations (Fig. 1d).

Among 683 ctDNA-positive cases, patients with BC exhibited the highest mean-VAF, while OC showed the lowest (Fig. 1e). Mean-VAF mirrored cfDNA concentration trends across clinical stages, tumor sizes, and disease statuses (Fig. 1f–h).

The overall ctDNA detection sensitivity was 61.5%, with cancer-specific sensitivities ranging from 30.0% (OC) to 89.2% (PC). Sensitivity increased with advanced stage (*r* = 0.955, *p* = 0.045), larger tumor diameter (*r* = 0.955, *p* = 0.045), poor treatment response (*r* = 0.900, *p* = 0.100), and higher cfDNA concentration (Fig. 1i–l, Supplementary Figs. 2, 3). As the minimum required mutation threshold was increased from 1 to 2 and subsequently to 3, a corresponding progressive decline in overall sensitivity was observed, decreasing from 61.7% to 52.0% and then to 46.0% (Supplementary Figs. 4).

### Concordance of Mutated Genes between Tissue and ctDNA

**Fig. 2:**
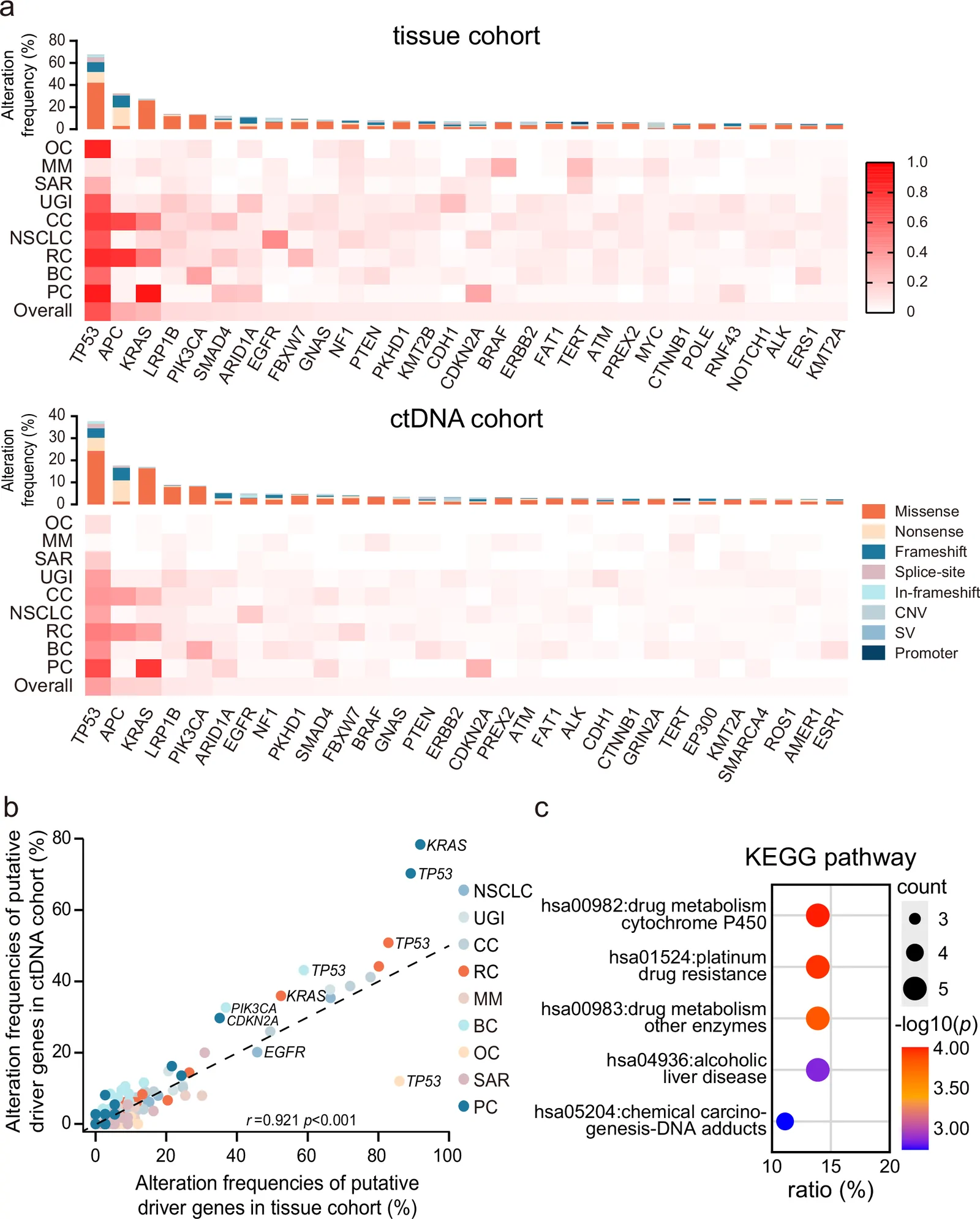
Mutational landscape and the mutational frequency of driver genes in both ctDNA and tissue cohorts, and pathway-based disruption signatures of tissue-specific mutated genes. **a** The heatmap illustrates the top 30 frequently encountered mutated genes in tissue and ctDNA cohorts. The accompanying vertical bars on the right depict the diverse mutational types prevalent in each gene. The gradation of colors adorning this diagram portrays the prevalence of these genetic aberrations across various cancer types. **b** The correlation between mutational frequency of driver genes in ctDNA and tissue cohorts. NSCLC non-small cell lung cancer, UGI upper gastrointestinal cancer, CC colon cancer, RC rectal cancer, MM melanoma, BC breast cancer, OC ovarian cancer, SAR sarcoma, PC pancreatic cancer. **c** The top five pathways of co-targets based on Kyoto Encyclopedia of Genes and Genomes (KEGG) enrichment analysis.

We performed comparative genomic profiling of paired tissue and plasma ctDNA samples to assess concordance of mutated genes. Initial analysis revealed strong concordance between tissue and plasma ctDNA profiles (Fig. 2a). The five most frequently altered genes showed remarkable consistency across both biospecimen types, with cancer-type-specific mutation patterns aligned with established genomic landscapes^12^. For example, *EGFR* mutations predominated in NSCLC (tissue: 45.7% vs. plasma: 20.1%), while UGI showed high rates of *CDH1* (26.6% vs. 11.6%), *ARID1A* (22.7% vs. 9.7%), and *LRP1B* (20.8% vs. 15.5%) mutations^3^. PC, CC, and RC displayed frequent *KRAS* alterations (91.9% vs. 78.4%, 49.0% vs. 25.5%, and 52.5% vs. 35.9%, respectively)^13^, whereas MM exhibited distinct *BRAF* (30.2% vs. 7.9%) and *TERT* (25.4% vs. 7.9%) single nucleotide variant (SNV) profiles^14^. BC demonstrated similar *PIK3CA* mutation rates between biospecimens (36.5% vs. 32.6%)^15^.

Pathway-level analysis (Supplementary Fig. 5) categorized mutated genes into 11 core oncogenic pathways. DNA damage repair (DDR), receptor tyrosine kinase (RTK)-RAS, and p53 pathways emerged as pan-cancer hotspots, contrasting with WNT pathway predominance in colorectal malignancies. Notably, MM showed infrequent p53 pathway disruption (14/63), while BC exhibited frequent PI3K-AKT activation (63.2% vs. 65.3%) compared to rare occurrences in PC (10.8% vs. 18.9%). Pathway dysregulation patterns demonstrated high tissue-plasma concordance across tumor types.

Subsequently, three mutated gene categories were delineated: 370 genes with mutations concordantly detected in both tissue and ctDNA, 36 tissue-specific mutated genes, and a single ctDNA-specific mutated gene (*MLLT1*). Correlation analysis of the frequencies of putative driver gene revealed robust linear agreement between biospecimens (*r* = 0.921, *p* < 0.001, Fig. 2b). Plasma mutation frequencies generally approximated 50% of tissue levels, with exceptions including reduced *TP53* detection in ovarian cancers (12.0% vs. 86.0%) and elevated *TP53* (89.2% vs. 70.3%) and *KRAS* (91.9% vs. 78.4%) rates in pancreatic malignancies.

Encyclopedia of Genes and Genomes (KEGG) analysis of co-mutations revealed significant enrichment in drug metabolism (adjusted *p* = 9.86 × 10^−^^5^) and platinum drug resistance pathways (adjusted *p* = 1.10 × 10^−4^, Fig. 2c), complemented by GO findings implicating response to amino acid mechanisms (FDR = 0.016, *p* = 1.61 × 10^−4^; Supplementary Fig. 6).

### Tumor Mutational Burden (TMB) and Microsatellite Instability (MSI)

TMB-high/ blood TMB (bTMB) -high (≥10 mut/Mb) prevalence was lower in PC, SAR, OC, and MM (Supplementary Fig. 7). The proportion of bTMB-high was higher than that of TMB-high only in BC patients. MSI- high occurred in 40 patients, distributed across UGI (*n* = 15), CC (*n* = 19), RC (*n* = 4), and BC (*n* = 2). All MSI-H cases exhibited tissue TMB-high, yet only 32.5% (13/40) demonstrated concurrent bTMB-high.

### Distribution of Clinically Actionable Alterations

All genomic alterations were annotated using OncoKB (2024 release), identifying 966 (7.8%) tissue-derived and 530 (10.2%) ctDNA-derived variants with therapeutic relevance (Supplementary Fig. 8). Level 1 alterations (FDA-approved biomarkers) showed comparable prevalence in paired biospecimens, accounting for 19.9% (192/966) of tissue and 19.6% (104/530) of ctDNA variants.

Actionable alterations were detected in 77.1% (857/1,111) of tissue and 49.2% (547/1,111) of plasma samples. Nearly all colorectal (98.4% of CC and 98.3% of RC) and 93.3% of NSCLC patients harbored tissue-based actionable alterations, whereas OC (34.0%) and SAR (21.8%) cohorts exhibited lower rates of clinically informative variants (Supplementary Fig. 9). Compared to tissue, ctDNA demonstrated significantly reduced actionable alteration frequencies in NSCLC (40.9%), MM (19.0%), OC (4.0%), and SAR (5.5%). Notably, plasma ctDNA maintained higher concordance with tissue findings in CC (70.0%), RC (80.1%), BC (55.8%), and PC (78.4%), with observed detection rates demonstrating less pronounced disparity between liquid and tissue biopsy approaches (Supplementary Fig. 10).

### Mutational clonality dynamics in ctDNA and tissue reveal subclonal enrichment patterns

**Fig. 3:**
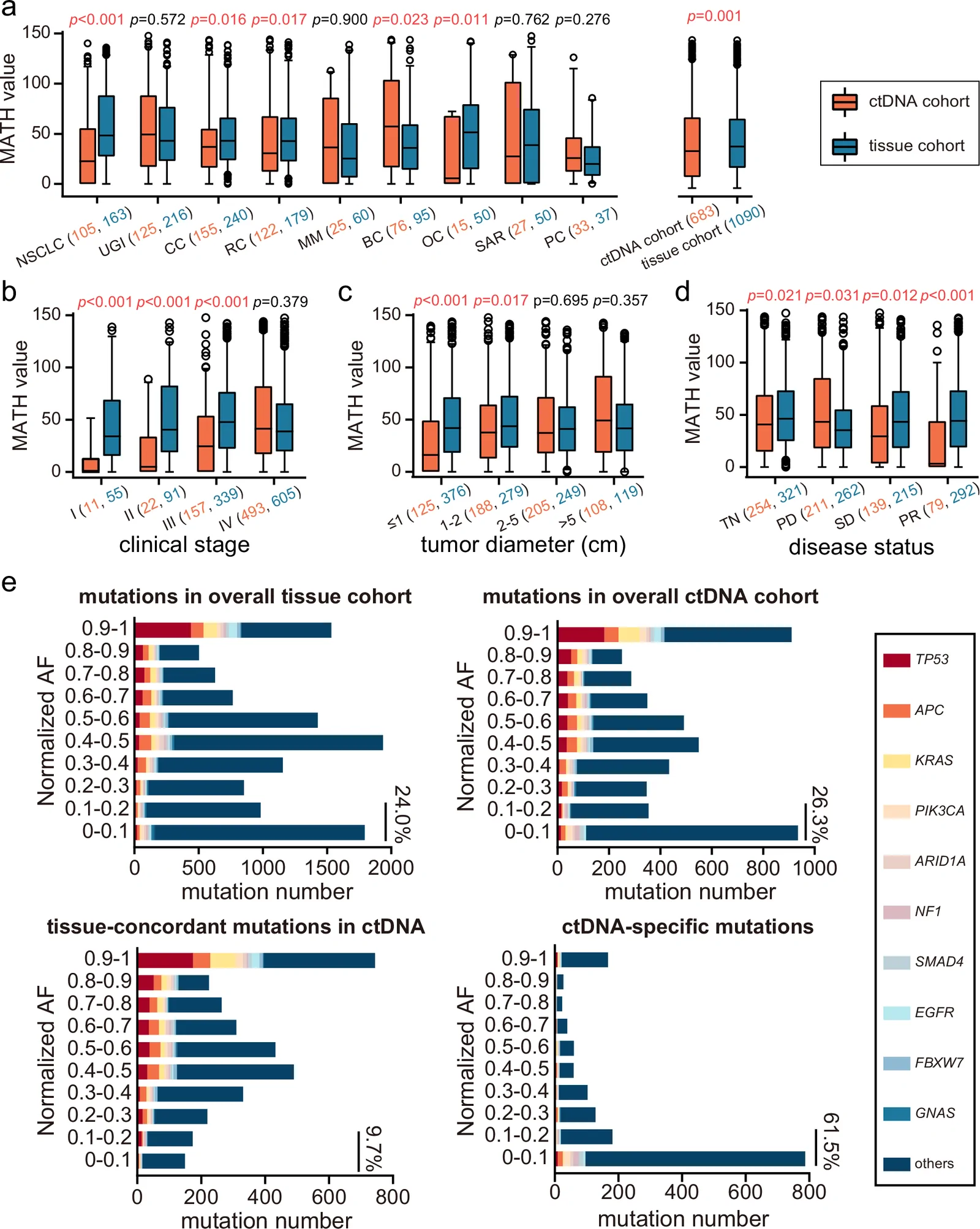
Mutational clonality analysis in ctDNA and tissue using MATH and normalized AF. The MATH values were analyzed for different cancer types (**a**), clinical stage (**b**), tumor diameter (**c**), and disease statuses (**d**) between the ctDNA and tissue cohorts using a two-sided Mann-Whitney U test. The numbers in red and blue correspond to the sample sizes in the ctDNA and tissue cohorts, respectively. Centre line within boxes, median; box limits, upper and lower quartiles; whiskers, 5–95% percentile; rings, outliers. Comparisons of clonality between ctDNA-specific mutations and tissue-concordant mutations were performed using normalized AF (**e**). Several common mutant genes were depicted in various colors, illustrating shifts in their fractions across different clonality ranges. The percentage in the bottom right corner of each image represents the proportion of clonality within the subgroup of 0–0.2 compared to all clonality ranges. MATH mutant-allele tumor heterogeneity, AF allele frequencies, NSCLC non-small cell lung cancer, PD progressive disease, SD stable disease, CR complete response, PR partial response.

After excluding samples without detectable variants, clonality analysis included 683 plasma and 1090 tissue samples. The median MATH value was significantly lower in ctDNA than in tissue (37.1 vs. 41.7, *p* = 0.001; Fig. 3a), with cancer-type-specific patterns: NSCLC, CC, RC, and OC showed reduced ctDNA-MATH values compared to tissue, whereas BC displayed a striking inverse trend: ctDNA-MATH higher than tissue-MATH. Early-stage (I–III) tumors and smaller lesions (<2 cm diameter) exhibited markedly lower ctDNA-MATH than tissue-MATH (Fig. 3b, c). Conversely, PD correlated with elevated ctDNA-MATH relative to tissue (*p* = 0.031), contrasting with higher tissue-MATH values in other disease statuses (Fig. 3d).

Normalized allele frequencies (AFs) revealed bimodal clonality distributions in both ctDNA and tissue, with mutations clustering at either high (>0.9) or low (<0.1) normalized AF ranges (Fig. 3e). Variants with normalized AF > 0.9 were enriched for canonical driver genes, representing 54.3% of the top ten drivers in tissue and 45.8% in ctDNA. In contrast, variants with normalized AF < 0.1 showed diminished driver representation (8.6% in tissue vs. 12.0% in ctDNA).

Defining subclonal mutations as those with normalized AF < 0.2, ctDNA harbored a marginally higher proportion of subclonal variants than tissue (26.3% vs. 24.0%). Strikingly, tissue-concordant mutations in ctDNA predominantly occupied the high- normalized AF range (>0.9), with only 9.7% classified as subclonal. Conversely, ctDNA-specific mutations peaked in the low normalized AF range (<0.1), with 61.5% originating from subclones (Fig. 3e). Driver genes constituted 34.7% of tissue-concordant mutations but only 14.0% of ctDNA-specific variants, underscoring the latter’s subclonal predominance.

Cancer-type stratification revealed NSCLC tissue samples carried the highest subclonal burden (34.8%), which decreased sharply ‌ in matched ctDNA (26.5%). BC exhibited increased subclonal representation in ctDNA compared to tissue (43.7% vs. 20.5%), while PC showed minimal subclonal variants in both biospecimens (Supplementary Fig. 11). Subclonal fractions in ctDNA escalated with advanced staging, larger tumor size, and poorer treatment response (Supplementary Figs. 12–14). However, the same trend was absent in tissue.

### Concordance of somatic alterations between tissue and ctDNA and influencing factors

Tissue sequencing identified 12,350 somatic alterations (9482 SNVs, 1920 indels, 768 CNVs, 180 SVs). After excluding 16 patients without detectable tissue mutations, the cohort exhibited an average of 11.12 mutations per patient. Comparative ctDNA analysis identified 5204 alterations (4091 SNVs, 732 indels, 294 CNVs, 87 SVs), with 429 plasma samples showing no detectable mutations (mean: 4.68 mutations per sample).

**Fig. 4:**
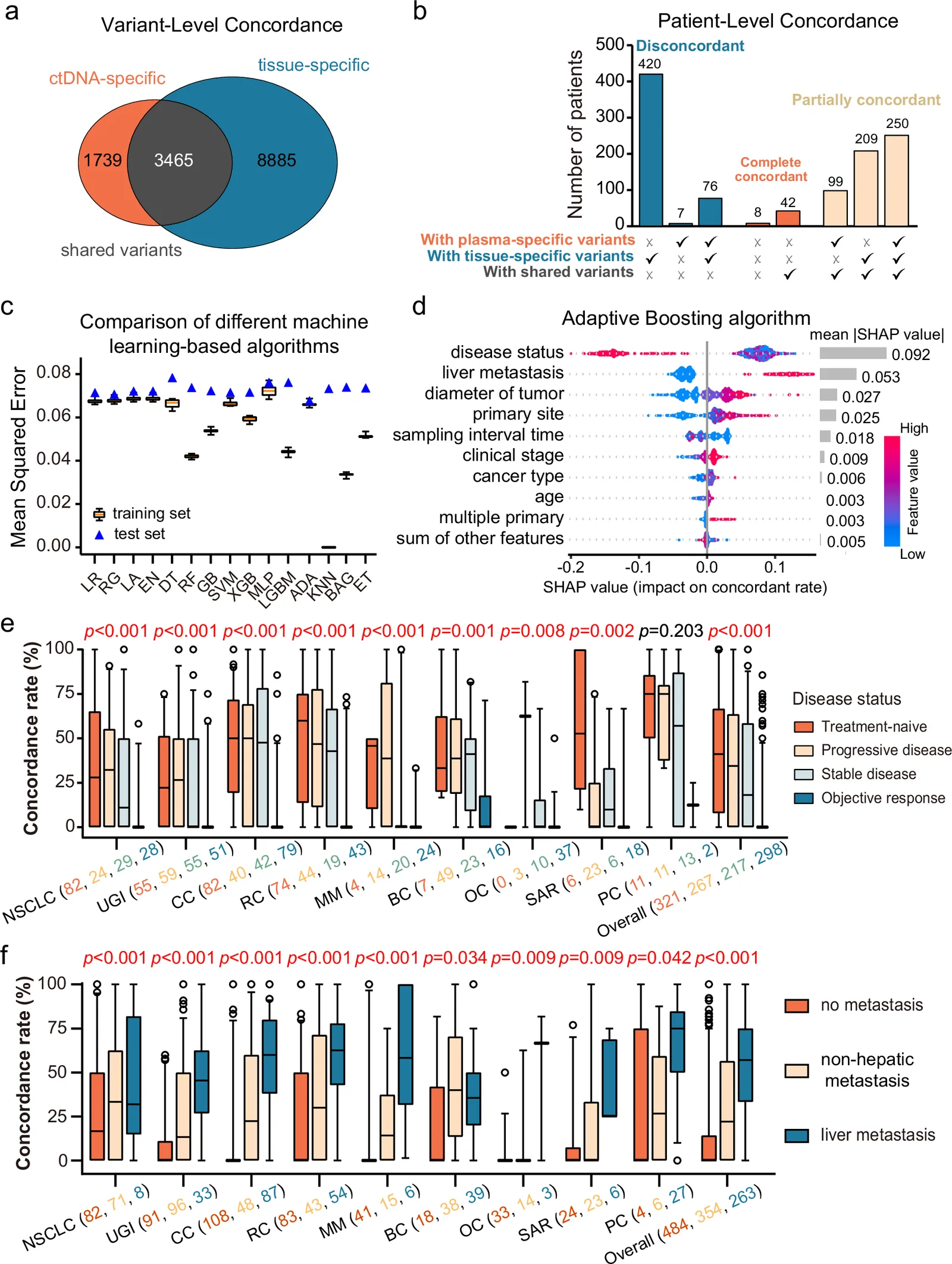
Concordance of somatic alterations between tissue and ctDNA cohorts and the relevant influencing factor analysis. **a** Variant-level concordance between paired tissue and ctDNA showing the number of genetic mutations, ctDNA-specific, tissue-specific, and tissue-concordant. **b** Patient-level concordance showing the number of patients in discordant, completely concordant, and partially concordant groups. **c** Fifteen machine-learning models were developed and evaluated by the mean squared error (MSE). Box diagrams and triangles represent the MSE of the training set and test set, respectively. **d** The model with the lowest MSE value in the test set was selected. SHAP explainer was created using the loaded machine learning models, and the SHAP values are calculated for the test set. Group comparisons of concordance rate among patients with different disease statuses (**e**) and liver metastasis statuses (**f**) in each cancer type and the overall cohort. Centre line within boxes, median; box limits, upper and lower quartiles; whiskers, 5–95% percentile; asterisk, outliers.

To assess pairwise concordance, we categorized 14,089 somatic variations into three groups: 8885 tissue-specific, 1739 ctDNA-specific, and 3465 shared variants (Fig. 4a), yielding an overall concordance rate of 24.6%. Patients were stratified by variant distribution: 45.3% discordant (mutations exclusive to one biospecimen), 4.5% completely concordant, and 50.2% partially concordant (Fig. 4b). As shown in Supplementary Table 2, ctDNA-specific mutations per patient gradually increased with advancing clinical stage and poorer treatment response, whereas tissue-specific mutations peaked at stage II (18.89) and declined in later stages. Notably, patients achieving CR/PR harbored the highest level of tissue-specific mutation burden (11.15/patient) but the lowest ctDNA-specific mutations (0.32/patient). Tissue-centric and plasma-centric concordance was shown in Supplementary Table 3 and 4, respectively.

To elucidate determinants of concordance, we trained 15 machine-learning models. The adaptive boosting algorithm (AdaBoost) demonstrated optimal performance with the lowest validation mean squared error (MSE; Fig. 4c). SHAP analysis identified disease status, liver metastasis, and tumor diameter as the top predictors of concordance (Fig. 4d), a ranking consistent across all models (Supplementary Fig. 15).

Cancer-type stratified analysis revealed that improved treatment response (Fig. 4e) and smaller tumor diameter (Supplementary Fig. 16) correlated with significantly reduced concordance rates in all cancers except PC, which did not reach statistical significance. Liver metastasis was associated with significantly higher concordance, though this trend did not reach statistical significance in NSCLC and BC (Supplementary Fig. 17). To address confounding by non-hepatic metastases, patients without liver metastasis were subdivided into those with metastases to other organs and those without distant metastasis. Concordance rates followed a descending gradient: liver metastasis > non-hepatic metastasis > no metastasis (Fig. 4f). Subsequent evaluation of liquid biopsy performance metrics demonstrated coordinated biological gradients across these clinical subgroups. Plasma cfDNA concentration, mean-VAF, and ctDNA detection sensitivity all mirrored the hierarchical progression observed in concordance rates. Patients with hepatic metastases exhibited maximal values across all three parameters, followed by non-hepatic metastasis cases, while non-metastatic patients showed minimal biomarker values (Supplementary Fig. 18).

### Association of ctDNA Status with Overall Survival

**Fig. 5:**
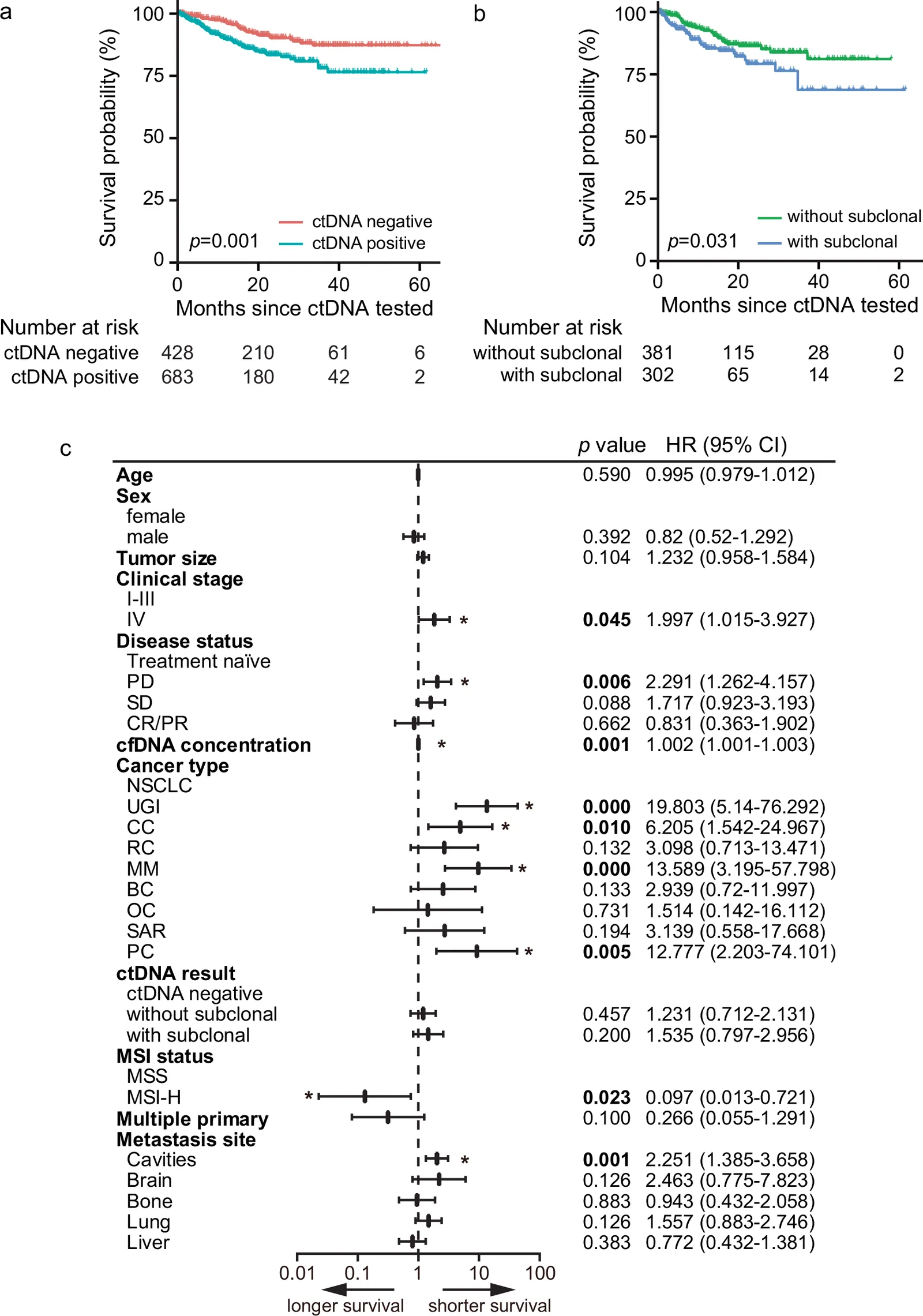
Univariate and multivariate analysis of overall survival. Kaplan-Meier estimates of overall survival (OS) stratified on detectable of mutations in ctDNA (**a**) and detectable of subclonal mutations in ctDNA (**b**). Results of multivariate of COX regression of putative risk factors influencing OS and the calculated hazard ratio (HR) and 95% confidence interval (CI) (**c**). PD progressive disease, SD stable disease, CR complete response, PR partial response, NSCLC non-small cell lung cancer, UGI upper gastrointestinal cancer, CC colon cancer, RC rectal cancer, MM melanoma, BC breast cancer, OC ovarian cancer, SAR sarcoma, PC pancreatic cancer, microsatellite stability (MSS); microsatellite instability-high (MSI-H).

As of February 26, 2025, the median follow-up duration was 13.2 months (interquartile range: 3.3–24.2 months). Univariate analysis revealed significantly shorter overall survival (OS) in ctDNA-positive patients compared to ctDNA-negative counterparts (hazard ratio [HR] = 1.999, 95% confidence interval [CI]: 1.348 – 2.966; *p* < 0.001; Fig. 5a). Among ctDNA-positive patients, those harboring subclonal mutations exhibited poorer OS than those without subclonal variants (HR = 1.653 95%CI: 1.016–2.691; *p* = 0.031; Fig. 5b).

Multivariate Cox regression confirmed advanced clinical stage (HR = 1.997, 95% CI: 1.015–3.927; *p* = 0.045), PD (HR = 2.291, 95% CI = 1.262–4.147; *p* = 0.006), elevated cfDNA concentration (HR = 1.002 per unit increase; 95% CI: 1.001–1.003; *p* = 0.001), and metastasis to the chest, abdominal, or pelvic cavities (HR = 2.251; 95% CI: 1.385–3.658; *p* = 0.001) as independent predictors of shorter OS. Conversely, microsatellite instability-high (MSI-H) status was associated with markedly prolonged survival (HR = 0.097; 95% CI: 0.013–0.721; *p* = 0.023). Compared to NSCLC patients, those with UGI, CC, MM, or PC demonstrated significantly shorter OS (Fig. 5c). Due to the MSI-H events were predominantly observed in gastrointestinal malignancies (Supplementary Table 1), we conducted a focused survival analysis exclusively for gastrointestinal malignancies. Results showed that MSI-H status remained a significant independent favorable prognostic factor for gastrointestinal tumors (HR = 0.087, 95%CI: 0.011–0.666, *p* = 0.019, Supplementary Fig. 19).

## Discussion

The genomic discordance between ctDNA and tumor tissue profiles presents a major obstacle to the clinical implementation of liquid biopsies. Our pan-cancer analysis of 1111 patients addresses this challenge through a multidimensional approach integrating mutational clonality assessment, machine learning, and clinical correlations. We demonstrate that ctDNA-tissue discrepancies arise from fundamental biological processes, particularly subclonal heterogeneity and organ-specific ctDNA dynamics. These findings redefine ctDNA as both a biomarker of tumor burden and a dynamic indicator of clonal heterogeneity, offering critical insights for precision oncology.

The predominance of subclonal variants in ctDNA-specific mutations (61.5%) compared to tissue-concordant variants (9.7%) aligns with models positing that hematogenous dissemination of subclones precedes metastatic colonization^16,17^. This subclonal enrichment, coupled with reduced representation of driver genes (14.0% in ctDNA-specific vs. 34.7% in tissue-concordant variants), highlights ctDNA’s unique ability to detect clusters associated with treatment resistance and poor prognosis. Notably, the survival disadvantage linked to ctDNA subclonality (HR = 1.653) suggests liquid biopsies could enhance risk stratification beyond traditional binary classification of ctDNA as positive or negative^18,19^. Conversely, tissue-specific mutations were enriched in drug metabolism pathways, likely reflecting spatially restricted, treatment-resistant niches. While tissue outperformed ctDNA in detecting actionable alterations (77.1% vs. 49.2%), ctDNA demonstrated superior resolution of subclonal architecture. This dichotomy underscores their complementary roles: tissue remains indispensable for initial biomarker discovery, particularly for therapies targeting canonical drivers (e.g., *EGFR* in NSCLC), whereas ctDNA excels in tracking clonal dynamics and monitoring evolving resistance mechanisms during treatment.

Technical strategies to improve ctDNA sensitivity, such as increasing sequencing depth^20^, optimized library conversion efficiency^21^, and fragment size analysis^22^, should consider biological determinants of ctDNA release and degradation^23^. Although clinical stage and tumor size are established predictors of ctDNA detectability, which have been well known as tumor burden indicators^9,24,25^, our machine learning framework uniquely identifies liver metastasis as a key concordance driver independent of tumor burden. This phenomenon may stem from impaired hepatic clearance of ctDNA, as the liver is a primary site of cfDNA degradation^26^. Reducing the phagocytosis of cfDNA by liver macrophages or inhibiting cfDNA degradation in peripheral blood through antibody-bound nucleosomes could increase cfDNA concentration, thereby improving ctDNA sensitivity^27^. Supporting this, patients with liver metastases exhibited elevated cfDNA concentrations, variant allele frequencies, and detection sensitivity in this study, aligning with murine models demonstrating reduced cfDNA turnover during hepatic dysfunction^26^. Notably, Bando et al. reported that colorectal cancer patients with metastatic in lung or peritoneal metastases have significantly lower concordance rates than those with liver metastases, further corroborating our findings. In this study, we expand prior associations between hepatic dysfunction to elevated cfDNA levels and increased concordance to a pan-cancer context.

The 24.6% overall ctDNA-tissue concordance rate underscores the interplay of technical and biological complexity. Prior studies, often limited to driver genes, single clinical stage, or single cancer types, may overestimate concordance due to narrower genomic scope. Our analysis of 437 genes across nine malignancies reveals that methodological differences in concordance calculations are primary contributors to reported variability. For example, our variant-level approach contrasts with gene-centric methods, inherently yielding lower concordance rates. This methodological rigor provides a more nuanced benchmark for future studies aiming to harmonize liquid and tissue biopsy applications.

This study’s retrospective design and uneven cancer-type distribution limit generalizability. While our 437-gene panel balances cost and coverage, whole-genome sequencing may better resolve subclonal diversity. Future work should integrate spatial transcriptomics to map subclone-microenvironment interactions and serial liquid biopsies to track clonal trajectories during malignant therapies.

In summary, this study delineates ctDNA’s dual role: a sensitive detector of subclonal heterogeneity and a prognostic biomarker, yet a suboptimal standalone tool for initial biomarker discovery. By elucidating how metastatic sites and treatment modulate ctDNA-tissue discordance, we provide a framework for optimizing liquid biopsy use in precision oncology. These insights advocate for a hybrid diagnostic approach, where tissue guides initial therapy and ctDNA monitors resistance, ultimately advancing personalized cancer management.

## Methods

### Study Design and Participants

This retrospective study analyzed paired plasma and tumor tissue samples from 1,111 patients diagnosed with nine solid tumor types (non-small cell lung cancer [NSCLC], upper gastrointestinal cancer [UGI], colon cancer [CC], rectal cancer [RC], melanoma [MM], breast cancer [BC], ovarian cancer [OC], sarcoma [SAR], and pancreatic cancer [PC]). Patients were consecutively enrolled at Peking University Cancer Hospital & Institute between September 2019 and October 2023. Clinical data included age, sex, smoking/drinking history, tumor diameter, clinical stage (according to the American Joint Committee on Cancer [AJCC] 8th edition), primary/metastatic sites, and disease status at the time of plasma sample collection (treatment-naïve [TN], progressive disease [PD], stable disease [SD], partial response [PR] or complete response [CR] according to the Response Evaluation Criteria in Solid Tumors [RECIST] version 1.1). Tumor diameter was determined via imaging, surgery, or endoscopy; post-resection diameters were recorded as 0 cm. This study was conducted in accordance with the Declaration of Helsinki and approved by the Medical Ethics Committee of Peking University Cancer Hospital & Institute (approval no. 2016XJS01). All experiments were carried out with the understanding and written informed consent of each participant.

### DNA Extraction and Next-generation Sequencing

All samples were tested in a Clinical Laboratory Improvement Amendments (CLIA), International ISO 15189, College of American Pathologists (CAP), and European Molecular Genetics Quality Network (EMQN) certified genomic testing facility (Nanjing Geneseeq Technology Inc.). Peripheral blood (10 mL) was collected in Streck cfDNA tubes and centrifuged at 2500 × *g* (10 min) and 16,000 × *g* (10 min) to isolate plasma and matched leukocytes. DNA was extracted using Qiagen kits: QIAamp Circulating Nucleic Acid Kit (plasma), QIAamp DNA Blood Mini Kit (leukocytes), and QIAamp DNA FFPE Tissue Kit (tissue). DNA quantification utilized Picogreen fluorescence assays (Invitrogen) with lambda DNA standards.

All samples, including tumor tissues, matched leukocytes, and plasma-derived cfDNA, were uniformly subjected to targeted sequencing using the same 437-gene pan-cancer panel (Nanjing Geneseeq Technology Inc.; with the full gene list available in Supplementary Table 5). Hybrid capture-based libraries were prepared according to the manufacturer’s protocols and sequenced on Illumina HiSeq 4000 platforms. Microsatellite instability status was assessed exclusively through tissue-based analysis.

### Bioinformatics analysis and cutoff value determination

All samples were tested in a Clinical Laboratory Improvement Amendments (CLIA), International ISO 15189, College of American Pathologists (CAP), and European Molecular Genetics Quality Network (EMQN) certified genomic testing facility (Nanjing Geneseeq Technology Inc.). All testing operations were carried out in accordance with the standardized operating procedures (SOP) of the testing company. Base calling was performed on sequencing reads in FASTQ format using bcl2fastq (version 2.19.0.316). Quality control, including the removal of terminal adapter sequences and low-quality segments, was conducted with Trimmomatic (version 0.36). The cleaned reads were then aligned to the human reference genome (hg19, GRCh37 Genome Reference Consortium Human ref.^37^) using the Burrows-Wheeler Aligner (BWA, version 0.7.12) with default parameters. Duplicate reads were identified and realigned to correct for mismatches and recalibrate base quality scores, utilizing the Genome Analysis Toolkit (version 3.2).

Somatic single nucleotide variants (SNVs) and short insertions/deletions (indels) were detected using Vardict, with a minimum variant allele frequency threshold set at 0.01. Fusions were identified using DELLY^28^with a minimum split read threshold of 3. Copy number variants (CNVs) were analyzed using CNVkit^29^, analyzing copy number values adjusted by sample ploidy. Cutoffs were defined as a fold change ≥1.6 for gains and ≤0.6 for losses.

### Germline and clonal hematopoiesis-related variants filtration

To ensure the high confidence of somatic variant calls in plasma cfDNA, a multi-step filtration strategy was employed to remove potential germline and clonal hematopoiesis (CHIP)-related variants. First, all candidate alterations detected in cfDNA were systematically matched against variants identified in paired genomic DNA (gDNA) from peripheral blood leukocytes, which were sequenced to a median depth of 200×. Variants present in the gDNA were classified as either germline or CHIP-derived and were excluded from subsequent somatic analyses.

To further enhance the accuracy of CHIP variant detection, we employed a machine learning-based classifier that integrates multiple genomic features, including variant allele fraction, population frequency, cfDNA fragmentation profiles, and single-base substitution signatures, building upon established methodologies^30,31^. Putative CH-associated variants were additionally filtered using an in-house population database comprising 4544 patients with diverse solid tumors as previously described^32^.

To minimize false positives resulting from sequencing artifacts and background noise, a combination of molecular and computational error-suppression techniques was applied. This included a custom background error model constructed using sequencing data from 500 white blood cell controls and plasma samples from 100 healthy individuals. Additionally, zero-inflated Poisson statistical modeling^33^ and intra-sample contextual error suppression^34^ were implemented to accurately characterize and subtract technical noise.

### Automated Triple Groom Sequencing (ATG-Seq) analysis pipeline

To enable sensitive and specific detection of low-abundance mutations in cell-free DNA (cfDNA), we employed Automated Triple Groom Sequencing (ATG-Seq) – an ultra-high-sensitivity ctDNA detection technology independently developed by Geneseeq^8^. The integrated molecular tagging strategy incorporates a dual-index barcoding system during library construction: i) unique molecular identifiers (UMIs) tagging individual DNA fragments, and ii) sample-specific barcodes. This dual-index approach allows precise tracing of sequencing reads back to their original DNA molecules and biological samples, significantly enhancing detection accuracy. ATG-Seq combines proprietary sequencing adapters with single-molecule tagging. By analyzing molecular tags and alignment positions in sequencing data, the platform reconciles single-strand and duplex reads originating from identical DNA templates. This core innovation substantially reduces technical noise from DNA damage, PCR amplification artifacts, and sequencing errors.

To assemble a position- and base substitution-specific background error database based on allele frequency and distinct supporting reads throughout the panel, we constructed a bioinformatics polishing pipeline by sequencing a pool of plasma samples collected from over 30 healthy donors. An alteration was considered as sequencing noise if its allele frequency and distinct supporting reads were not significantly higher than the corresponding background errors in the database. To minimize the errors from PCR, hybridization, damaging, sequencing, and contamination, and avoid mutations from non-tumor sources in cfDNA, we conducted the following procedures: (i) we sequenced the cfDNA fragment at a depth of ~5000× (before deduplication), which produced redundant DNA molecules; (ii) mapping positions and a dual-barcode system were used to maximize the representative power of unique DNA molecules; (iii) a duplex assisted decoder system was used to filter mapping and sequencing artifacts. Our approach utilizes mutation-specific detection criteria calibrated to our CLIA-certified sequencing platform, requiring: (i) ≥ 3 supporting reads at ≥ 100 × coverage for ctDNA variants also detected in matched tissue; (ii) ≥ 6 supporting reads at ≥ 100 × coverage for ctDNA variants absent in tissue. A sample is classified as ctDNA-positive upon detection of at least one mutation meeting these stringent requirements. Complete quality control metrics were provided in the Supplementary Tables 6 and 7, respectively.

### Gene Ontology (GO) and Kyoto Encyclopedia of Genes and Genomes (KEGG) signaling pathway analyses

To elucidate the function of tissue-specific mutated genes, the database of Annotation, Visualization, and Integrated Discovery (DAVID, https://david.ncifcrf.gov/) was used to analyze the enrichment in Gene Ontology (GO) functions and Kyoto Encyclopedia of Genes and Genomes (KEGG) signaling pathways. Tissue-specific mutated genes involved in the cellular components (CC), molecular functions (MF), biological processes (BP), and pathways were described. We selected the top 20 GO terms and the top 5 KEGG pathways with the smallest *p*-value and imported them into R 4.3.2 to draw bubble graphs and bar graphs.

### Mutational clonality and heterogeneity analysis

Mutant-allele tumor heterogeneity (MATH) was calculated as 148.26 × (median absolute deviation [MAD]/median) of allele frequencies (AFs) for each sample^35^.

Normalized AFs were derived by first adjusting for copy number variations (transformed AF = AF/log2 (copy number)); for copy-neutral/loss regions, AFs remained unadjusted. Normalized AFs were scaled to the sample’s maximum transformed AF (range: 0–1)^12^. Variants with normalized AF below 0.2 were classified as subclonal^36^.

### Clinically Actionable Variants

Therapeutic relevance of mutations was annotated using OncoKB (2024 release)^37,38^. There are four levels for sensitive markers and two levels for resistance markers. Level 1 was defined as an FDA-recognized biomarker predictive of response to an FDA-approved drug in corresponding cancer types. Level 2 was defined as a standard-care biomarker recommended by the NCCN or other professional guidelines. Level 3 was defined as a variation with compelling clinical evidence supporting its function as a predictive biomarker of drug response. Level 4 was defined as a variation with compelling biological evidence supporting its function as a predictive biomarker of drug response. Resistance level 1 was defined as a standard care biomarker predictive of resistance to an FDA-approved drug. Resistance level 2 was defined as a variation with compelling clinical evidence supporting its function as a predictive biomarker of resistance to a drug. When a patient carried multiple gene mutations related to treatment at different levels of evidence, the highest level of evidence was recorded. CC and RC are exceptions when analyzing the potentially actionable alterations. The absence of mutation in *KRAS* and *NRAS* is clinically relevant as RAS mutation status influences patient responses to the anti-EGFR antibody therapies cetuximab and panitumumab. When *KRAS* and *NRAS* mutations were not detected in both plasma and tissue, it was recorded as therapeutic level 1: FDA-approved drugs. When *KRAS* or *NRAS* mutations were detected in either tissue or plasma, the sample with mutations was recorded as resistance level 1, and the other sample without mutations was considered false negative and recorded as wild-type (samples without any alterations) or other alterations’ levels rather than FDA-approved drugs level. TMB or blood TMB (bTMB) ≥ 10 mut/Mb was defined as TMB-High and recorded as therapeutic level 1.

### Variant-Level Concordance Analysis

We use the Jaccard Index to calculate the concordance rate, and the formula is as follows:

Concordance rate = shared variants/(shared variants + tissue-specific variations + ctDNA-specific variations)^9^. Eight patients without detectable variants in both samples were excluded.

Tissue-centric concordance = shared variants/(shared variants + tissue-specific variations).

Plasma-centric concordance = shared variants/(shared variants + ctDNA-specific variations).

### Machine learning models development and comparison

The “mice” (Multivariate Imputation by Chained Equations) package in the R programming language was used to handle missing data by generating multiple imputations for each missing value. “Standard Scaler” package in Python was used to data scaling. Subsequently, clinical data were divided, with 80% utilized for training and 20% for validation. A total of 17 features, including age, sex, smoke, drink, clinical stage, tumor size, disease status, ratio of tumor cells in tissue sample, cfDNA concentration, interval time between the tissue biopsy and the blood draw, cancer type, primary site, multiple primaries, brain metastasis, bone metastasis, cavities metastasis, lung metastasis, were used to develop the models. Fifteen machine learning models, namely, liner regression, ridge regression, LASSO regression, elastic net regression, decision tree, random forest, gradient boosting, support vector machine, extreme gradient boosting, light gradient boosting machine, multilayer perceptron, adaptive boosting, K-nearest neighbors, bootstrap aggregating, extra randomized trees, were used to predict the concordance ratio of genetic alterations in tissue and ctDNA and the concentration of cfDNA. In order to optimize the prediction model, grid search combined with manual fine-tuning was applied to obtain the final hyperparameters.

Several common evaluation indexes, such as the mean squared error (MSE), root mean squared error (RMSE), mean absolute error (MAE), and R-squared, were used to evaluate the reliability of these models. In addition, ten-fold cross-validation was conducted on the training set for the validation of the model.

### Feature importance explanation

The SHapley Additive Explanation (SHAP) method is an approach that could rank the importance of input features and explain the results of the prediction model, and it is implemented to overcome the “black-box” issue. After adjusting the hyperparameters and developing the machine learning models, the SHAP explainer was created using the loaded machine learning models, and the SHAP values were calculated for the test set. The global explanation could give consistent attribution values for each feature within a model to show the associations between input features and cfDNA concentration or concordance rate.

### Statistical analysis

Categorical variables were analyzed using *χ*² or Fisher’s exact tests as appropriate. Continuous variables were expressed as median values with 5th–95th percentiles, compared through Mann-Whitney U tests for pairwise comparisons or Kruskal-Wallis H tests and post hoc Dunn’s testing for multi-group analyses. Bivariate correlations between continuous parameters were assessed using Pearson coefficients. For patient-level ctDNA detection sensitivity, a true positive (TP) is defined as a positive test result in both the ctDNA sample and the matched tissue sample. Cases where ctDNA yielded negative results while tissue testing identified genetic variants were designated as false negatives (FN). Sensitivity was calculated using the formula: Sensitivity = TP/(TP + FN) × 100%. Overall survival outcomes were evaluated through Kaplan-Meier methodology, with statistical significance between groups assessed via Gehan-Breslow-Wilcoxon tests. Multivariate Cox proportional hazards models derived hazard ratios (HRs). All analyses were performed in R 4.3.2, Python 3.12.7, and GraphPad Prism 9, with statistical significance defined as two-sided *p* < 0.05.

## Supplementary information

revised Supplementary clean

## Data Availability

The datasets used and/or analyzed during the current study are available from the corresponding author on reasonable request.

## Code availability

The code is available from the corresponding author upon reasonable request.
