# Supplementary material for: Pan-cancer analysis of tissue-plasma genomic concordance reveals enhanced concordance with liver metastasis and plasma clonality as predictor of poorer overall survival
Source: NPJ Precis Oncol. 2026 Jul 3;10:407. doi: 10.1038/s41698-026-01549-0 (PMC13710981; doi:10.1038/s41698-026-01549-0)
Supplement: Supplementary file 1 [file 41698_2026_1549_MOESM1_ESM.pdf]

**Supplemental Information**

*Article title: Pan-Cancer Analysis of Tissue-Plasma Genomic Concordance Reveals Enhanced Concordance with Liver Metastasis and Plasma Clonality as Predictor of Poorer Overall Survival*


*Author names:* Meng Zhang[1], Yi Feng[1], Xue Du[1], Changda Qu[1], Meizhu Meng[1], Meiying Ye[1], Min Liang[1], Ziran Yang[1], Wenjuan Gong[1], Xingyu Ma[1], Jialiang Guo[1], Wenmei Li[1], Chenxuan Wang[2], Qiuxiang Ou[2], Sha Wang[2], Hua Bao[2], Shuqin Jia[1,*]

*Affiliation and e-mail address of the corresponding author:* Professor Shuqin Jia, Center for Molecular Diagnostics, State Key Laboratory of Holistic Integrative Management of Gastrointestinal Cancers, Beijing Key Laboratory of Carcinogenesis and Translational Research, Peking University Cancer Hospital & Institute, 52 Fucheng Road, Haidian District, Beijing, 100142, China. Tel: +86-010-88196926; Fax: +86-010-88122437; E-mail: jiashuqin2014@163.com

**Check list**

**Supplementary Tables**

Supplementary Table 1 Patient clinical characteristics.
Supplementary Table 2 Patients with different clinical stages and disease statuses carried ctDNA-specific and tissue-specific mutations.
Supplementary Table 3 Tissue-centric concordance across different cancer types.
Supplementary Table 4 Plasma-centric concordance across different cancer types.
Supplementary Table 5 The 437 targeted-panel gene list
Supplementary Table 6 Sequencing metric quality control summary across different sample types.
Supplementary Table 7 cfDNA sequencing metric quality control summary across different cancer types.

**Supplementary Figures**

Supplementary Fig. 1 The histogram of interval-time between the tissue biopsy and the blood draw.
Supplementary Fig. 2 The difference in cfDNA concentration between patients with ctDNA positive and negative result.
Supplementary Fig. 3 The correlation between cfDNA concentration and ctDNA sensitivity.
Supplementary Fig. 4 Trend of ctDNA sensitivity changes under different threshold conditions across different cancer types.
Supplementary Fig. 5 Spider web diagrams showed pathway-based disruption signatures in all patients and each of the nine cancer types.
Supplementary Fig. 6 The top 20 pathways of co-targets based on Gene Ontology (GO) enrichment analysis.
Supplementary Fig. 7 The proportion of TMB-High and bTMB-High in different cancer types.
Supplementary Fig. 8 Distribution of clinical actionable alterations in tissue and ctDNA.
Supplementary Fig. 9 The proportion of patients harboring potentially actionable alteration in tissue.
Supplementary Fig. 10 The proportion of patients harboring potentially actionable alteration in ctDNA.
Supplementary Fig. 11 Estimation of clonality among patients with different cancer types in the tissue and ctDNA cohorts.
Supplementary Fig. 12 Estimation of clonality among patients with different clinical stages in the tissue and ctDNA cohorts.
Supplementary Fig. 13 Estimation of clonality among patients with different tumor diameters in the tissue and ctDNA cohorts.
Supplementary Fig. 14 Estimation of clonality among patients with different disease status in the tissue and ctDNA cohorts.
Supplementary Fig. 15 SHAP summary bar plot of 15 machine learning models.
Supplementary Fig. 16 The effect of tumor diameter on the concordance of somatic variations between ctDNA and tissue among different cancer types.

Supplementary Fig. 17 Group comparisons of concordance rate among patients with different liver metastasis statuses in each cancer type and the overall cohort.

Supplementary Fig. 18 The concentration of cfDNA extracted from plasma, mean variant allele frequency (mean-VAF) of mutations in ctDNA, and detection sensitivity of ctDNA varied across different liver metastasis statuses.

Supplementary Fig. 19 Gastrointestinal-specific multivariate survival analysis

**Supplementary Tables**

**Supplementary Table 1 Patient clinical characteristics.**

| Characteristics | All | BC | CC | NSCLC | MM | OC | PC | RC | SAR | UGI |
|---|---|---|---|---|---|---|---|---|---|---|
| **Number of patients** | 1111 | 95 | 243 | 164 | 63 | 50 | 37 | 181 | 55 | 223 |
| **Age group** | | | | | | | | | | |
| <60 | 561 | 59 | 109 | 60 | 38 | 31 | 19 | 88 | 39 | 118 |
| ≥60 | 550 | 36 | 134 | 104 | 25 | 19 | 18 | 93 | 16 | 105 |
| **Sex** | | | | | | | | | | |
| Female | 501 | 95 | 107 | 58 | 28 | 50 | 14 | 52 | 21 | 76 |
| Male | 610 | 0 | 136 | 106 | 35 | 0 | 23 | 129 | 34 | 147 |
| **Smoke** | | | | | | | | | | |
| No | 651 | 88 | 145 | 60 | 37 | 46 | 21 | 98 | 32 | 124 |
| Yes | 323 | 2 | 67 | 80 | 17 | 2 | 7 | 59 | 7 | 82 |
| Unknown | 137 | 5 | 31 | 24 | 9 | 2 | 9 | 24 | 16 | 17 |
| **Alcohol** | | | | | | | | | | |
| No | 756 | 90 | 162 | 104 | 46 | 46 | 21 | 111 | 37 | 139 |
| Yes | 218 | 0 | 50 | 36 | 8 | 2 | 7 | 46 | 2 | 67 |
| Unknown | 137 | 5 | 31 | 24 | 9 | 2 | 9 | 24 | 16 | 17 |
| **Clinical stage** | | | | | | | | | | |
| I | 60 | 3 | 6 | 21 | 10 | 4 | 0 | 2 | 6 | 8 |
| II | 94 | 6 | 22 | 5 | 10 | 5 | 3 | 21 | 13 | 9 |
| III | 344 | 9 | 82 | 57 | 22 | 24 | 1 | 64 | 7 | 78 |
| IV | 613 | 77 | 133 | 81 | 21 | 17 | 33 | 94 | 29 | 128 |
| **Tumor size** | | | | | | | | | | |
| ≤1 cm | 388 | 26 | 99 | 34 | 37 | 38 | 4 | 52 | 24 | 74 |
| 1-2 cm | 283 | 16 | 65 | 29 | 6 | 5 | 4 | 75 | 5 | 78 |
| 2-5 cm | 251 | 35 | 46 | 56 | 13 | 5 | 17 | 30 | 5 | 44 |
| >5 cm | 119 | 10 | 22 | 32 | 2 | 1 | 10 | 17 | 12 | 13 |
| Unknown | 70 | 8 | 1 | 13 | 5 | 1 | 2 | 7 | 9 | 14 |
| **Disease status** | | | | | | | | | | |
| PD | 267 | 59 | 40 | 24 | 14 | 3 | 11 | 44 | 23 | 59 |
| SD | 219 | 23 | 42 | 29 | 21 | 10 | 13 | 19 | 6 | 56 |
| PR/CR | 304 | 16 | 79 | 29 | 24 | 37 | 2 | 44 | 20 | 53 |
| TN | 321 | 7 | 82 | 82 | 4 | 0 | 11 | 74 | 6 | 55 |
| **Ratio of tumor cells in tissue samples** | | | | | | | | | | |
| >20% | 805 | 77 | 197 | 104 | 53 | 32 | 21 | 126 | 49 | 146 |
| ≤20% | 297 | 18 | 46 | 52 | 10 | 18 | 16 | 55 | 6 | 76 |
| Unknown | 9 | 0 | 0 | 8 | 0 | 0 | 0 | 0 | 0 | 1 |
| **MSI status** | | | | | | | | | | |
| MSS | 1071 | 93 | 224 | 164 | 63 | 50 | 37 | 177 | 55 | 208 |
| MSI | 40 | 2 | 19 | 0 | 0 | 0 | 0 | 4 | 0 | 15 |
| **ctDNA status** | | | | | | | | | | |
| Negative | 428 | 19 | 88 | 59 | 38 | 35 | 4 | 59 | 28 | 98 |
| Positive | 683 | 76 | 155 | 105 | 25 | 15 | 33 | 122 | 27 | 125 |

BC, breast cancer; CC, colon cancer; NSCLC, non-small cell lung cancer; MM, melanoma; OC, ovarian cancer; PC, pancreatic cancer; RC, rectal cancer; SAR, sarcoma; UGI, upper gastrointestinal cancer; PD, progressive disease; SD, stable disease; PR, partial response; CR, complete response; TN, treatment naïve; MSS, microsatellite stability; MSI, microsatellite instability.

**Supplementary Table 2 Patients with different clinical stages and disease statuses carried ctDNA-specific and tissue-specific mutations.**

| | Number of patients | Number of ctDNA-specific mutations | ctDNA-specific mutations per patient | Number of tissue- specific mutations | tissue-specific mutations per patient |
|---|---|---|---|---|---|
| **Clinical stage** | | | | | |
| I | 60 | 10 (0.6%) | 0.17 | 431 (4.9%) | 7.18 |
| II | 94 | 20 (1.2%) | 0.21 | 1776 (20.0%) | 18.89 |
| III | 344 | 262 (15.1%) | 0.76 | 3894 (43.8%) | 11.32 |
| IV | 613 | 1447 (83.2%) | 2.36 | 2784 (31.3%) | 4.54 |
| **Disease status** | | | | | |
| TN | 321 | 592 (34.0%) | 1.84 | 2947 (33.2%) | 9.18 |
| PD | 267 | 785 (45.1%) | 2.94 | 1429 (16.1%) | 5.35 |
| SD | 219 | 265 (15.2%) | 1.21 | 1118 (12.6%) | 5.11 |
| PR/CR | 304 | 97 (5.6%) | 0.32 | 3391 (38.2%) | 11.15 |

TN, treatment naïve; PD, progressive disease; SD, stable disease; PR, partial response; CR, complete response

**Supplementary Table 3 Tissue-centric concordance across different cancer types.**

| Cancer types | Number of sample | Tissue-centric concordance Median (IQR) |
|---|---|---|
| BC | 95 | 50.0% (11.1%-80.0%) |
| CC | 240 | 25.0% (0.0%-80.0%) |
| NSCLC | 163 | 16.7% (0.0-57.1%) |
| MM | 61 | 0.0% (0.0%-36.7%) |
| OC | 50 | 0.0% (0.0%-0.0%) |
| PC | 37 | 80.0% (35.4%-100.0%) |
| RC | 180 | 50.0% (0.0%-33.3%) |
| SAR | 52 | 0.0% (0.0%-33.3%) |
| UGI | 218 | 0.0% (0.0%-60.0%) |

BC, breast cancer; CC, colon cancer; NSCLC, non-small cell lung cancer; MM, melanoma; OC, ovarian cancer; PC, pancreatic cancer; RC, rectal cancer; SAR, sarcoma; UGI, upper gastrointestinal cancer; IQR, interquartile range

**Supplementary Table 4 Plasma-centric concordance across different cancer types.**

| Cancer types | Number of samples | Tissue-centric concordance Median (IQR) |
|---|---|---|
| BC | 76 | 66.7% (44.7%-100.0%) |
| CC | 155 | 83.3% (60.0%-100.0%) |
| NSCLC | 105 | 96.9% (65.2%-100.0%) |
| MM | 25 | 60.0% (2.0%-100.0%) |
| OC | 15 | 0.0% (0.0-83.3%) |
| PC | 33 | 90.1% (75.0%-100.0%) |
| RC | 122 | 87.5% (64.1%-100.0%) |
| SAR | 27 | 66.7% (20.0%-100.0%) |
| UGI | 125 | 66.7% (30.6%-100.0%) |

BC, breast cancer; CC, colon cancer; NSCLC, non-small cell lung cancer; MM, melanoma; OC, ovarian cancer; PC, pancreatic cancer; RC, rectal cancer; SAR, sarcoma; UGI, upper gastrointestinal cancer; IQR, interquartile range

**Supplementary Table 5 The 437 targeted-panel gene list**

Single nucleotide variations (SNVs) and insertions/deletions (InDels) were identified in 437 genes

| | | | | | | |
|---|---|---|---|---|---|---|
| *ABCB1* | *ABCC2* | *ADGRB3* | *ADH1B* | *AFDN* | *AIP* | *AKT1* |
| *AKT2* | *AKT3* | *ALDH2* | *ALK* | *AMER1* | *APC* | *AR* |
| *ARAF* | *ARID1A* | *ARID1B* | *ARID2* | *ARID5B* | *ASCL4* | *ASXL1* |
| *ATF1* | *ATIC* | *ATM* | *ATR* | *ATRX* | *AURKA* | *AURKB* |
| *AXIN2* | *AXL* | *B2M* | *BAD* | *BAK1* | *BAP1* | *BARD1* |
| *BAX* | *BCL2* | *BCL2L11* | *BCR* | *BIRC3* | *BLM* | *BMPR1A* |
| *BRAF* | *BRCA1* | *BRCA2* | *BRD4* | *BRIP1* | *BTG2* | *BTK* |
| *BUB1B* | *CASP8* | *CBL* | *CBLB* | *CCN6* | *CCND1* | *CCNE1* |
| *CD274* | *CD74* | *CDA* | *CDC73* | *CDH1* | *CDK10* | *CDK12* |
| *CDK4* | *CDK6* | *CDK8* | *CDKN1A* | *CDKN1B* | *CDKN1C* | *CDKN2A* |
| *CDKN2B* | *CDKN2C* | *CEBPA* | *CEP57* | *CHD4* | *CHD8* | *CHEK1* |
| *CHEK2* | *CREBBP* | *CRKL* | *CSF1R* | *CTCF* | *CTLA4* | *CTNNB1* |
| *CUL3* | *CUX1* | *CXCR4* | *CYLD* | *CYP19A1* | *CYP2A13* | *CYP2A6* |
| *CYP2A7* | *CYP2B6*6* | *CYP2C19* | *CYP2C9*3* | *CYP2D6* | *CYP3A4*4* | *CYP3A5* |
| *CYSLTR2* | *DAXX* | *DDR2* | *DENND1A* | *DHFR* | *DICER1* | *DLL3* |
| *DNMT3A* | *DOT1L* | *DPYD* | *DTL* | *DUSP2* | *EGFR* | *EIF1AX* |
| *EML4* | *EMSY* | *EP300* | *EPAS1* | *EPCAM* | *EPHA2* | *EPHA3* |
| *EPHA5* | *ERBB2* | *ERBB3* | *ERBB4* | *ERBIN* | *ERCC1* | *ERCC2* |
| *ERCC3* | *ERCC4* | *ERCC5* | *ESR1* | *ETV1* | *ETV4* | *ETV5* |
| *ETV6* | *EWSR1* | *EXT1* | *EXT2* | *EZH2* | *EZR* | *FANCA* |
| *FANCC* | *FANCD2* | *FANCE* | *FANCF* | *FANCG* | *FANCI* | *FANCL* |
| *FANCM* | *FAT1* | *FBXW7* | *FGF19* | *FGFR1* | *FGFR2* | *FGFR3* |
| *FGFR4* | *FH* | *FLCN* | *FLT1* | *FLT3* | *FLT4* | *FOXA1* |
| *FOXL2* | *FOXO1* | *FOXP1* | *FRG1* | *GATA1* | *GATA2* | *GATA3* |
| *GATA4* | *GATA6* | *GNA11* | *GNAQ* | *GNAS* | *GREB1* | *GREM1* |
| *GRIN2A* | *GRM3* | *GRM8* | *GSTM1* | *GSTM4* | *GSTP1* | *GSTT1* |
| *HDAC2* | *HDAC9* | *HGF* | *HLA-A* | *HNF1A* | *HNF1B* | *HOXB13* |
| *HRAS* | *IDH1* | *IDH2* | *IFNA6* | *IFNB1* | *IFNE* | *IFNG* |
| *IFNGR1* | *IFNGR2* | *IGF1R* | *IGF2* | *IKBKE* | *IKZF1* | *IL7R* |
| *INPP4B* | *IRF2* | *JAK1* | *JAK2* | *JAK3* | *JARID2* | *JUN* |
| *KDM5A* | *KDR* | *KEAP1* | *KIF1B* | *KIT* | *KITLG* | *KLLN* |
| *KMT2A* | *KMT2B* | *KMT2C* | *KMT2D* | *KRAS* | *LHCGR* | *LMO1* |
| *LRP1B* | *LYN* | *LZTR1* | *MAP2K1* | *MAP2K2* | *MAP2K4* | *MAP3K1* |
| *MAP3K4* | *MAX* | *MC1R* | *MCL1* | *MDM2* | *MDM4* | *MECOM* |
| *MED12* | *MEF2B* | *MEN1* | *MET* | *MGMT* | *MITF* | *MLH1* |
| *MLH3* | *MLLT1* | *MLLT3* | *MPL* | *MRE11* | *MSH2* | *MSH6* |
| *MTAP* | *MTHFR* | *MTOR* | *MUTYH* | *MYBL1* | *MYC* | *MYCL* |
| *MYCN* | *MYD88* | *MYH9* | *NAT1* | *NBN* | *NCOR1* | *NF1* |

| | | | | | | |
|---|---|---|---|---|---|---|
| *NF2* | *NFE2L2* | *NFKBIA* | *NKX2-1* | *NOTCH1* | *NOTCH2* | *NOTCH3* |
| *NPM1* | *NQO1* | *NRAS* | *NRG1* | *NSD1* | *NTHL1* | *NTRK1* |
| *NTRK2* | *NTRK3* | *NUTM1* | *PAK3* | *PALB2* | *PALLD* | *PARP1* |
| *PARP2* | *PAX5* | *PBRM1* | *PDCD1* | *PDCD1LG2* | *PDE11A* | *PDGFRA* |
| *PDGFRB* | *PDK1* | *PGR* | *PHOX2B* | *PIK3C3* | *PIK3CA* | *PIK3CD* |
| *PIK3R1* | *PIK3R2* | *PKHD1* | *PLAG1* | *PLCB4* | *PLK1* | *PMS1* |
| *PMS2* | *POLD1* | *POLD3* | *POLE* | *POLH* | *POT1* | *PPARD* |
| *PPM1D* | *PPP2R1A* | *PPP2R2A* | *PRDM1* | *PREX2* | *PRF1* | *PRKACA* |
| *PRKAR1A* | *PRKCI* | *PRKDC* | *PRKN* | *PRSS1* | *PRSS3* | *PTCH1* |
| *PTEN* | *PTK2* | *PTPN11* | *PTPN13* | *QKI* | *RAC1* | *RAC3* |
| *RAD50* | *RAD51* | *RAD51B* | *RAD51C* | *RAD51D* | *RAD54L* | *RAF1* |
| *RARA* | *RARG* | *RASGEF1A* | *RB1* | *RECQL4* | *RELN* | *RET* |
| *RHBDF2* | *RHOA* | *RICTOR* | *RNF43* | *ROS1* | *RPTOR* | *RRM1* |
| *RUNX1* | *RUNX1T1* | *SBDS* | *SDC4* | *SDHA* | *SDHB* | *SDHC* |
| *SDHD* | *SEPTIN9* | *SETBP1* | *SETD2* | *SF3B1* | *SGK1* | *SKP2* |
| *SLC34A2* | *SLC3A2* | *SMAD2* | *SMAD3* | *SMAD4* | *SMAD7* | *SMARCA4* |
| *SMARCB1* | *SMO* | *SOCS1* | *SOS1* | *SOX2* | *SPOP* | *SPRED1* |
| *SPRY4* | *SRC* | *SRSF2* | *SRY* | *STAG2* | *STAT3* | *STK11* |
| *STMN1* | *SUFU* | *TACC3* | *TAP1* | *TAP2* | *TEK* | *TEKT4* |
| *TERC* | *TERT* | *TET2* | *TGFBR2* | *THADA* | *TMEM127* | *TMPRSS2* |
| *TNFAIP3* | *TNFRSF11A* | *TNFRSF14* | *TNFRSF19* | *TNFSF11* | *TOP1* | *TOP2A* |
| *TP53* | *TP63* | *TPMT* | *TSC1* | *TSC2* | *TSHR* | *TTF1* |
| *TUBB3* | *TYMS* | *U2AF1* | *UGT1A1* | *VAMP2* | *VEGFA* | *VHL* |
| *WAS* | *WRN* | *WT1* | *XPA* | *XPC* | *XRCC1* | *XRCC2* |
| *YAP1* | *ZNF217* | *ZNF703* | | | | |

Structural variations (SV) were identified in 57 genes

| | | | | | | |
|---|---|---|---|---|---|---|
| *AKT2* | *ALK* | *AXL* | *BCL2* | *BCR* | *BIRC3* | *BRAF* |
| *BRCA1* | *BRCA2* | *BRD4* | *CCND1* | *CD274* | *CD74* | *CDK12* |
| *CDK4* | *CDKN2A* | *EGFR* | *ERBB* | *ERBB3* | *ETV1* | *ETV4* |
| *ETV6* | *EWSR1* | *EZR* | *FGFR1* | *FGFR2* | *FGFR3* | *JAK2* |
| *KIT* | *KMT2A* | *MET* | *MYC* | *NOTCH1* | *NOTCH2* | *NPM1* |
| *NRG1* | *NTRK1* | *NTRK2* | *NTRK3* | *NUTM1* | *PDGFRA* | *PDGFRB* |
| *PRKACA* | *RAD50* | *RAF1* | *RARA* | *RELN* | *RET* | *ROS1* |
| *RUNX1* | *RUNX1T1* | *SDC4* | *SLC34A2* | *TACC3* | *TMPRSS2* | *TP53* |
| *YAP1* | | | | | | |

Copy number variations (CNVs) were identified in 103 genes

| | | | | | | |
|---|---|---|---|---|---|---|
| *ABCB1* | *AKT1* | *AKT2* | *AKT3* | *ALK* | *APC* | *AR* |
| *AURKB* | *AXL* | *BCL2* | *BMPR1A* | *BRAF* | *CCND1* | *CCNE1* |
| *CD274* | *CDH1* | *CDK12* | *CDK4* | *CDK6* | *CDKN2A* | *CDKN2B* |
| *CRKL* | *CSF1R* | *DDR2* | *DLL3* | *EGFR* | *EPCAM* | *ERBB2* |
| *ERBB3* | *ESR1* | *FGF19* | *FGFR1* | *FGFR2* | *FGFR3* | *FGFR4* |

| | | | | | | |
|---|---|---|---|---|---|---|
| *FLT1* | *FLT3* | *FLT4* | *GNAS* | *HGF* | *HRAS* | *IGF1R* |
| *IGF2* | *IL7R* | *JAK1* | *KIT* | *KRAS* | *MAP2K1* | *MAP2K2* |
| *MCL1* | *MDM2* | *MDM4* | *MET* | *MGMT* | *MLH1* | *MSH2* |
| *MSH6* | *MTOR* | *MUTYH* | *MYC* | *MYCN* | *NF1* | *NKX2-1* |
| *NOTCH2* | *NRAS* | *NTRK1* | *PDCD1LG2* | *PDGFRA* | *PDGFRB* | *PIK3CA* |
| *PMS2* | *POLD1* | *POLE* | *PTEN* | *PTK2* | *RB1* | *RET* |
| *RICTOR* | *RRM1* | *SDHA* | *SDHB* | *SDHC* | *SDHD* | *SMAD4* |
| *SMARCB1* | *SMO* | *SOX2* | *SRC* | *STK11* | *STMN1* | *TERC* |
| *TERT* | *TOP1* | *TOP2A* | *TP53* | *TSC1* | *TSC2* | *TTF1* |
| *TUBB3* | *VEGFA* | *VHL* | *ZNF217* | *ZNF703* | | |

**Supplementary Table 6 Sequencing metric quality control summary across different sample types.**

| | leukocyte | | plasma | | tissue | |
|---|---|---|---|---|---|---|
| | **Mean ± SD** | **Median (IQR)** | **Mean ± SD** | **Median (IQR)** | **Mean ± SD** | **Median (IQR)** |
| Raw reads | 3064476.6 ± 2093531.9 | 2815350 (2394506 - 3365279) | 46428432.8 ± 10825054.95 | 45288081 (39895584.5 - 51697143.5) | 12508285.02 ± 6689217.16 | 11398948 (8416223.5 - 15298301.5) |
| Clean reads | 2917689.47 ± 1952975.3 | 2692322 (2274833.5 - 3214007.5) | 44646835.88 ± 10593698.8 | 43648668 (38223151.5 - 49691266) | 11950102.1 ± 6478822.63 | 10855721 (8012745 - 14660634) |
| Raw bases | 930.98 ± 629.26 | 857 (728 - 1026) | 14056.49 ± 3333.21 | 13682 (12051 - 15675) | 3781.15 ± 2038.63 | 3433 (2521.5 - 4636.5) |
| GC content (%) | 46.8 ± 0.82 | 46.76 (46.32 - 47.21) | 48.73 ± 0.7 | 48.72 (48.36 - 49.06) | 48.58 ± 2.22 | 48.14 (47.145 - 49.7) |
| Q30 (%) | 91.75 ± 2.4 | 91.96 (90.795 - 93.035) | 92.98 ± 2.03 | 92.97 (91.88 - 94.05) | 92.44 ± 2.66 | 92.6 (91.26 - 93.79) |
| Insert size (bp) | 293 ± 28.94 | 299 (270 - 315) | 170.62 ± 4.64 | 171 (168 - 173) | 217.32 ± 45.45 | 213 (188 - 238) |
| Duplication rate (%) | 21.41 ± 8.28 | 20.7 (16.175 - 26.92) | 52.12 ± 11.56 | 52.19 (43.655 - 59.4) | 35.84 ± 15.75 | 31.97 (26.225 - 40.805) |
| Mapping rate (%) | 99.93 ± 0.04 | 99.93 (99.91 - 99.95) | 99.88 ± 0.06 | 99.88 (99.85 - 99.91) | 99.78 ± 1.56 | 99.91 (99.86 - 99.94) |
| Mean depth | 353.23 ± 793.54 | 298.94 (254.195 - 357.205) | 6346.88 ± 3882.41 | 5796.53 (5066.345 - 6646.805) | 1410.92 ± 774.81 | 1296.6 (929.75 - 1722.385) |
| Mean depth (post-deduplication) | 258.71 ± 257.24 | 234.01 (199.45 - 277.735) | 2840.82 ± 989.67 | 2664.25 (2218.785 - 3292.905) | 859.96 ± 441.37 | 816.8 (573.64 - 1083.975) |
| Median depth | 343.59 ± 806.59 | 289 (246 - 345) | 6266.84 ± 3955.76 | 5707 (4985 - 6501.5) | 1309.29 ± 738.95 | 1209 (855 - 1602) |
| Median depth (post-deduplication) | 251.7 ± 258.89 | 227 (193 - 269) | 2856.1 ± 988.55 | 2685 (2243 - 3304) | 807.13 ± 428.96 | 768 (536.5 - 1028.5) |
| On-target rate (%) | 80.76 ± 3.85 | 81.27 (79.615 - 82.87) | 88.3 ± 3.91 | 88.92 (87.84 - 89.84) | 83.1 ± 6.38 | 84.58 (81.17 - 86.605) |

**Supplementary Table 7 cfDNA sequencing metric quality control summary across different cancer types.**

| cancer types | cfDNA mass (ng) | Q30(%) | Mean depth | Mean depth (post-deduplication) | Median depth | Median depth (post-deduplication) | On-target rate (%) |
|---|---|---|---|---|---|---|---|
| BC | 90.8 (60.0-193.5) | 93.0 (92.0-94.2) | 5846.2 (5151.2-6588.0) | 2633.2 (2255.6-3364.7) | 5783 (5093-6444) | 2651 (2291.5-3291) | 89.1 (88.1-89.9) |
| CC | 77.3 (49.1-122.3) | 92.9 (92.0-94.1) | 5861.5 (5135.1-6568.6) | 2616.4 (2110.7-3177.2) | 5831.5 (5140-6594) | 2661 (2153-3212.5) | 88.8 (88.1-89.6) |
| MM | 52.1 (39.0-83.5) | 92.7 (91.5-94.1) | 5737.8 (4850.3-6847.3) | 2703.7 (2247.9-3693.8) | 5758 (4844-6834) | 2737 (2270-3750) | 89.1 (88.5-90.0) |
| NSCLC | 66.5 (44.4-101.4) | 92.9 (91.7-93.9) | 5801.0 (5084.4-6898.1) | 2716.6 (2329.3-3389.6) | 5743 (5008.5-6722.5) | 2766 (2358.5-3391.5) | 88.8 (87.5-89.8) |
| OC | 57.4 (36.3-83.0) | 93.2 (92.2-94.0) | 5740.9 (5065.6-6569.1) | 2750.5 (2262.1-3419.2) | 5705 (4949-6459.5) | 2725 (2279.5-3352.5) | 89.0 (88.1-89.8) |
| PC | 80.1 (60.5-177.0) | 93.0 (91.8-94.0) | 5664.9 (5075.7-6439.4) | 2487.6 (1892.8-2972.5) | 5666 (4982-6378.5) | 2509 (1947.5-3002.5) | 88.3 (87.3-89.5) |
| RC | 70.0 (43.4-115.4) | 92.8 (91.6-93.9) | 5760.7 (5036.8-6642.1) | 2698.1 (2213.2-3119.4) | 5687 (4971-6468) | 2705 (2240-3135) | 89.1 (88.0-89.9) |
| SAR | 57.7 (44.0-91.8) | 93.4 (92.5-94.3) | 5448.7 (4822.6-6068.3) | 2443.7 (1946.3-2789.9) | 5403 (4753-6040.5) | 2470 (1990.5-2803) | 88.8 (87.6-89.4) |
| UGI | 72.2 (48.7-111.2) | 92.8 (92.1-95.7) | 5897.4 (5088.5-7187.3) | 3038.1 (2547.2-3888.1) | 5697.5 (5050.3-7147.5) | 3072.5 (2586.5-3949.3) | 89.1 (88.1-90.2) |

**Supplementary Figures**

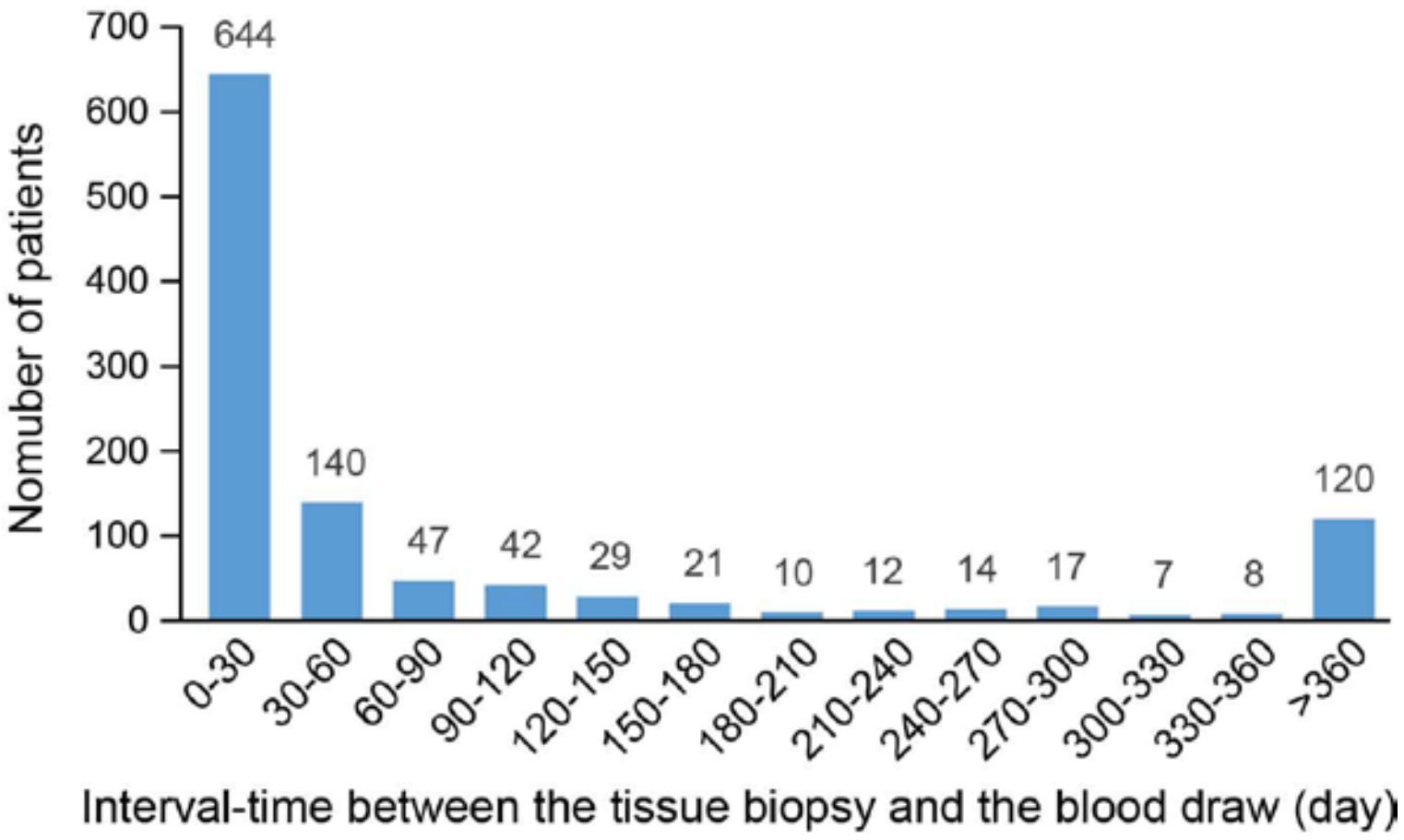


Supplementary Fig. 1 The histogram of interval-time between the tissue biopsy and the blood draw.

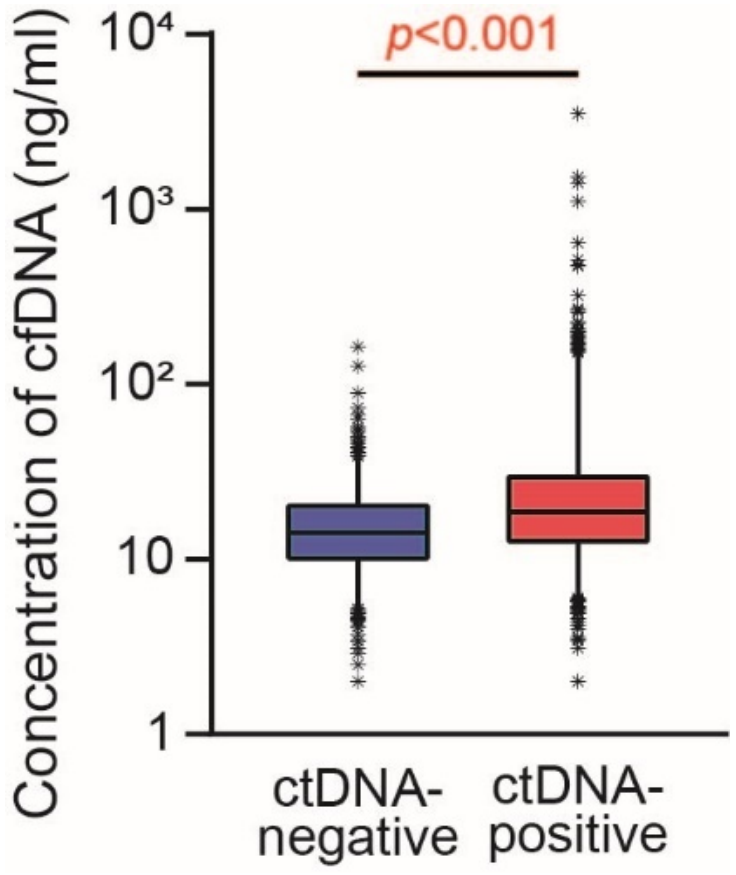


Supplementary Fig. 2 The difference in cfDNA concentration between patients with ctDNA positive and negative result. Centre line within boxes, median; box limits, upper and lower quartiles; whiskers, 5-95% percentile; asterisk, outliers.

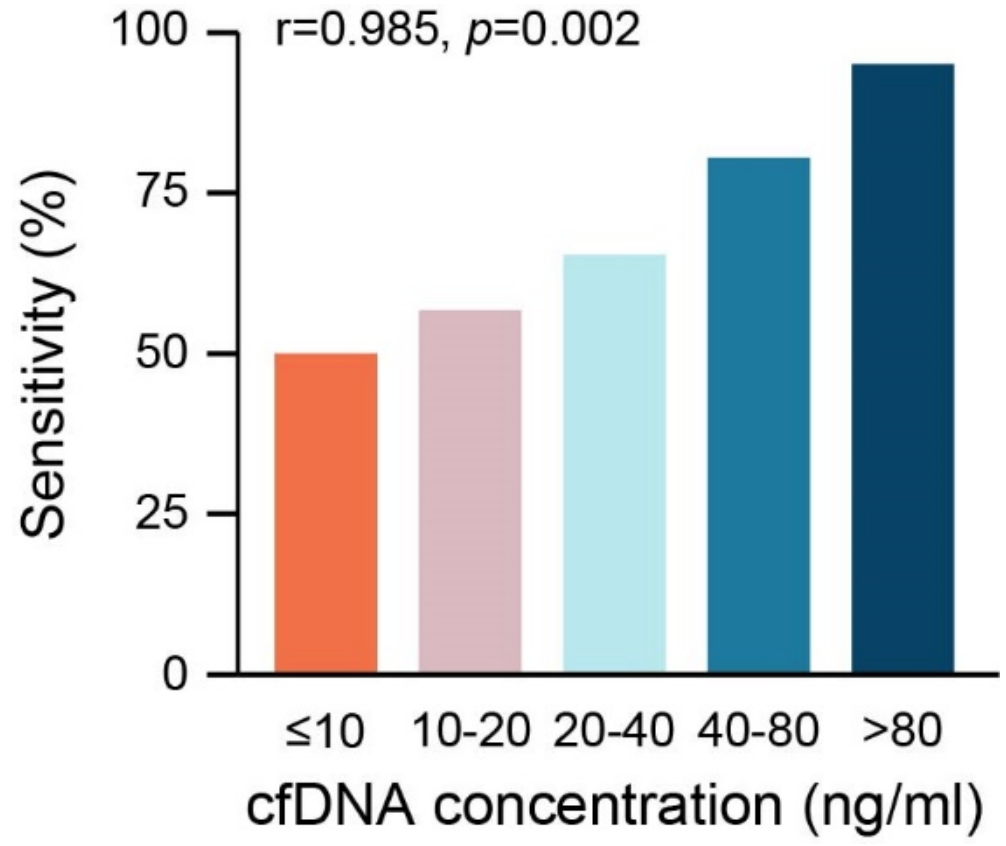

Supplementary Fig. 3 The correlation between cfDNA concentration and ctDNA sensitivity.

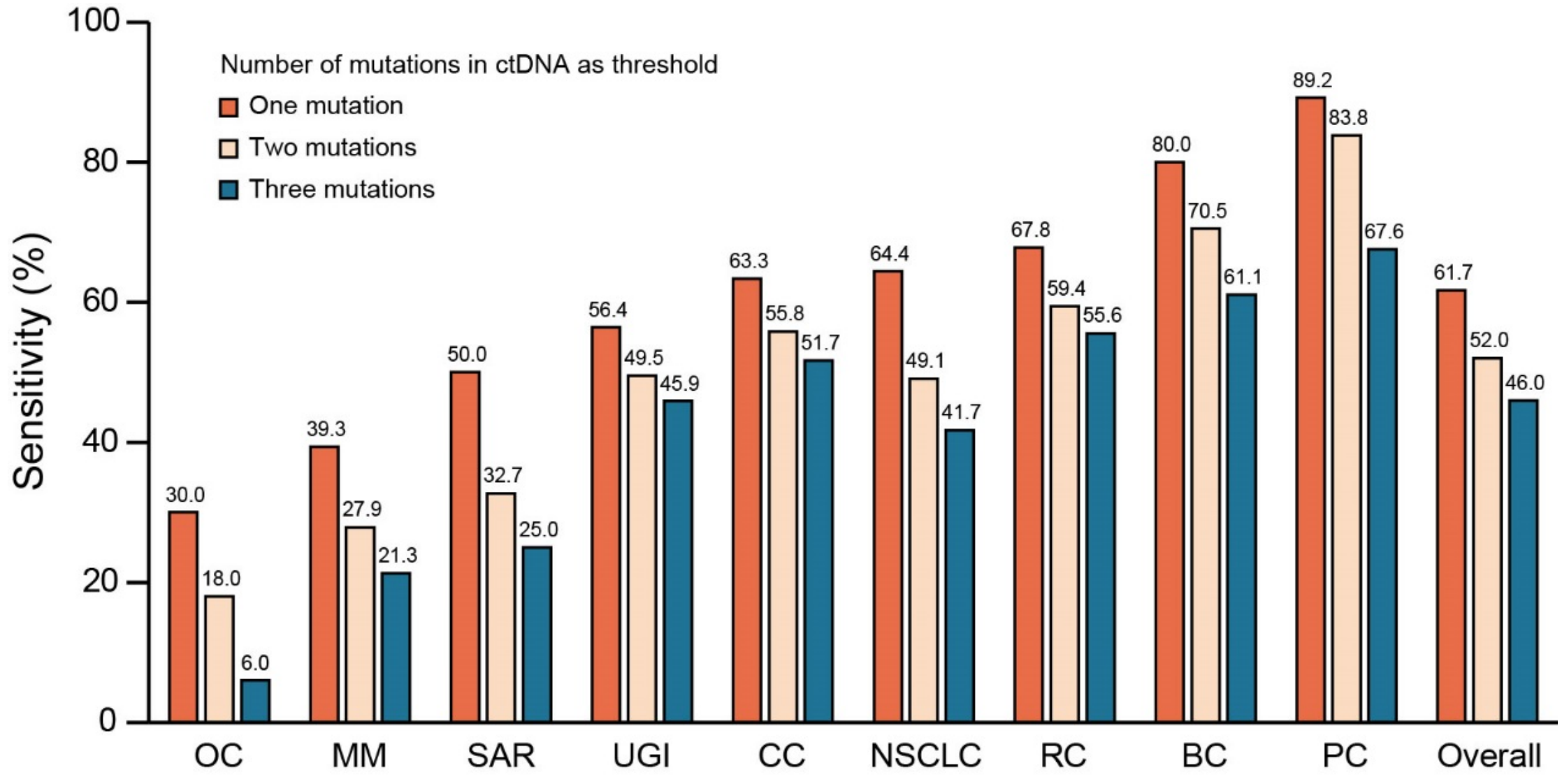


Supplementary Fig. 4 Trend of ctDNA sensitivity changes under different threshold conditions across different cancer types.

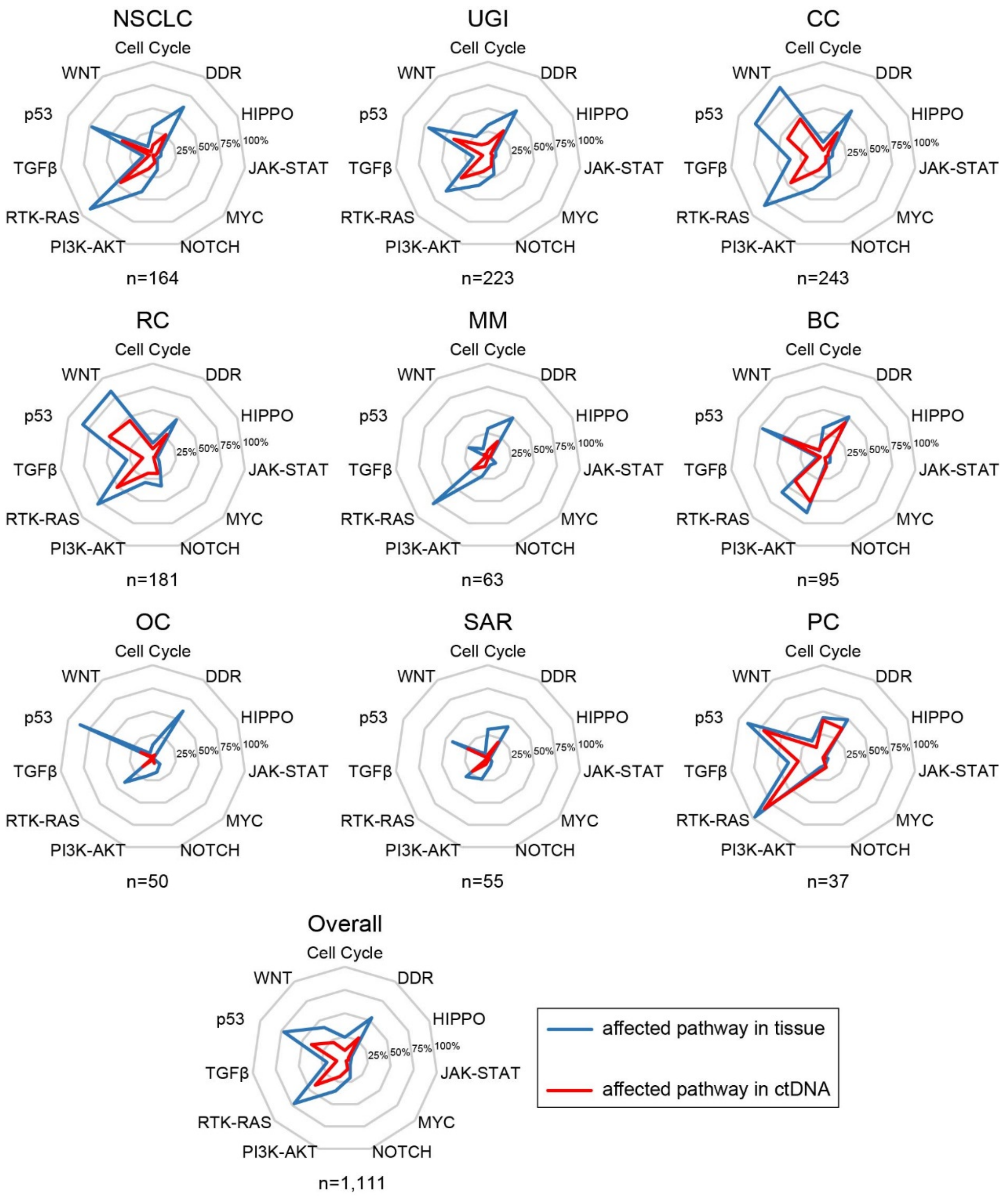


Supplementary Fig. 5 Spider web diagrams showed pathway-based disruption signatures in all patients and each of the nine cancer types. The lines in red and blue correspond to the disrupted pathway in the ctDNA and tissue cohorts, respectively. NSCLC, non-small cell lung cancer; UGI, upper gastrointestinal cancer; DDR, DNA damage repair; RTK, receptor tyrosine kinase; TGF, transforming growth factor.

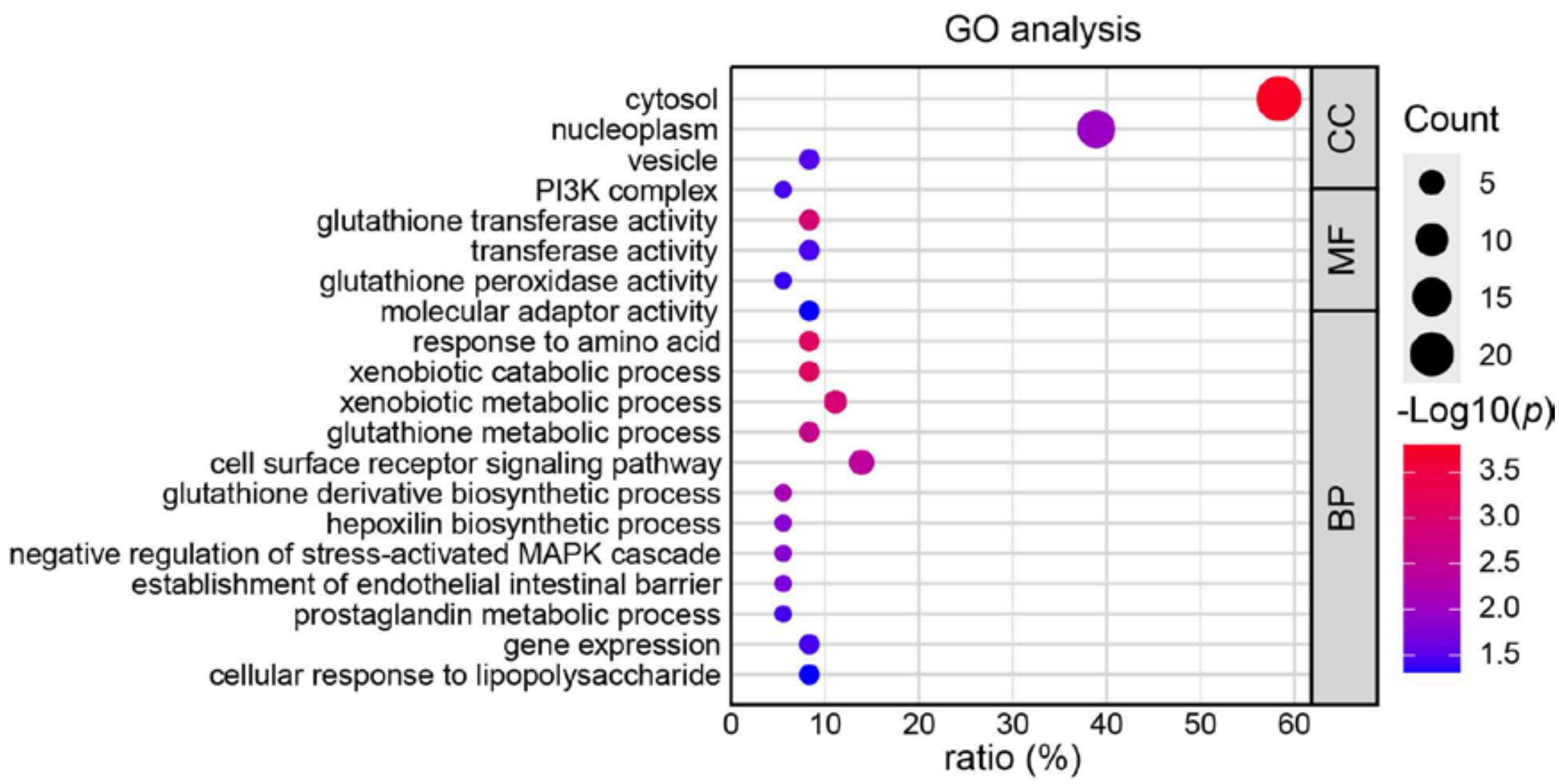


Supplementary Fig. 6 The top 20 pathways of co-targets based on Gene Ontology (GO) enrichment analysis. BP, biological processes ; CC, cellular components; MF, molecular functions.

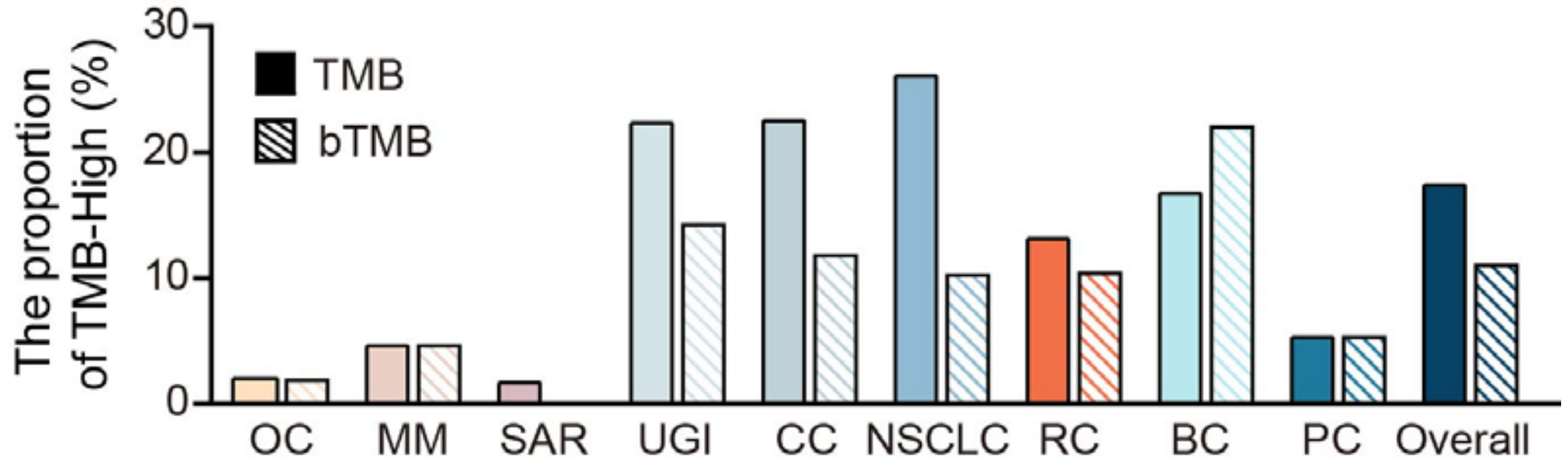


Supplementary Fig. 7 The proportion of TMB-High and bTMB-High in different cancer types. TMB, tumor mutation burden; bTMB, blood tumor mutation burden; NSCLC, non-small cell lung cancer; UGI, upper gastrointestinal cancer; CC, colon cancer; RC, rectal cancer; MM, melanoma; BC, breast cancer; OC, ovarian cancer; SAR, sarcoma; PC, pancreatic cancer.

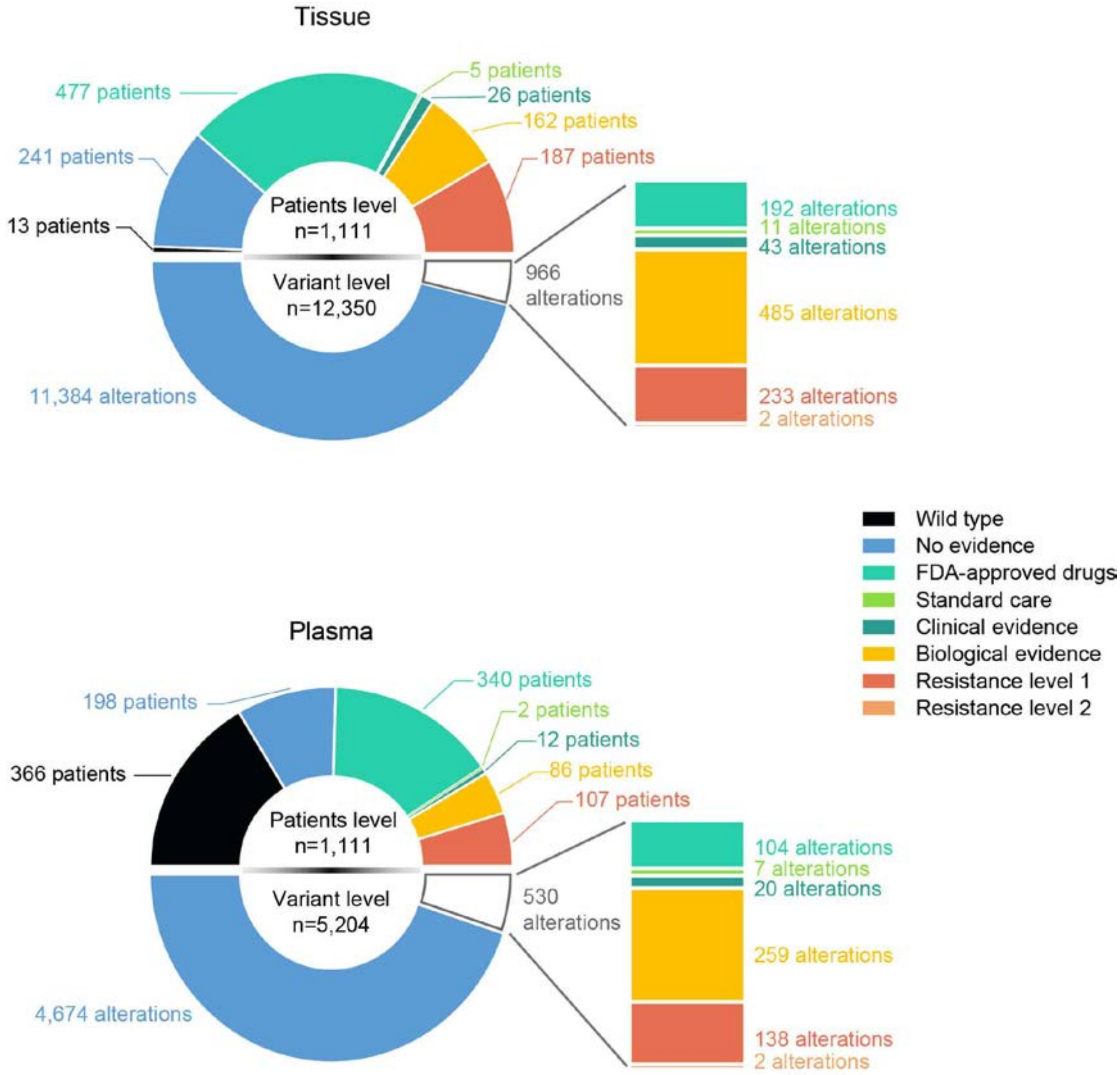


Supplementary Fig. 8 Distribution of clinical actionable alterations in tissue and ctDNA. The upper semicircle depicted the prevalence of patients harboring genomic alterations across various therapeutic levels. The lower semicircle illustrated the number of mutations at diverse therapeutic levels.

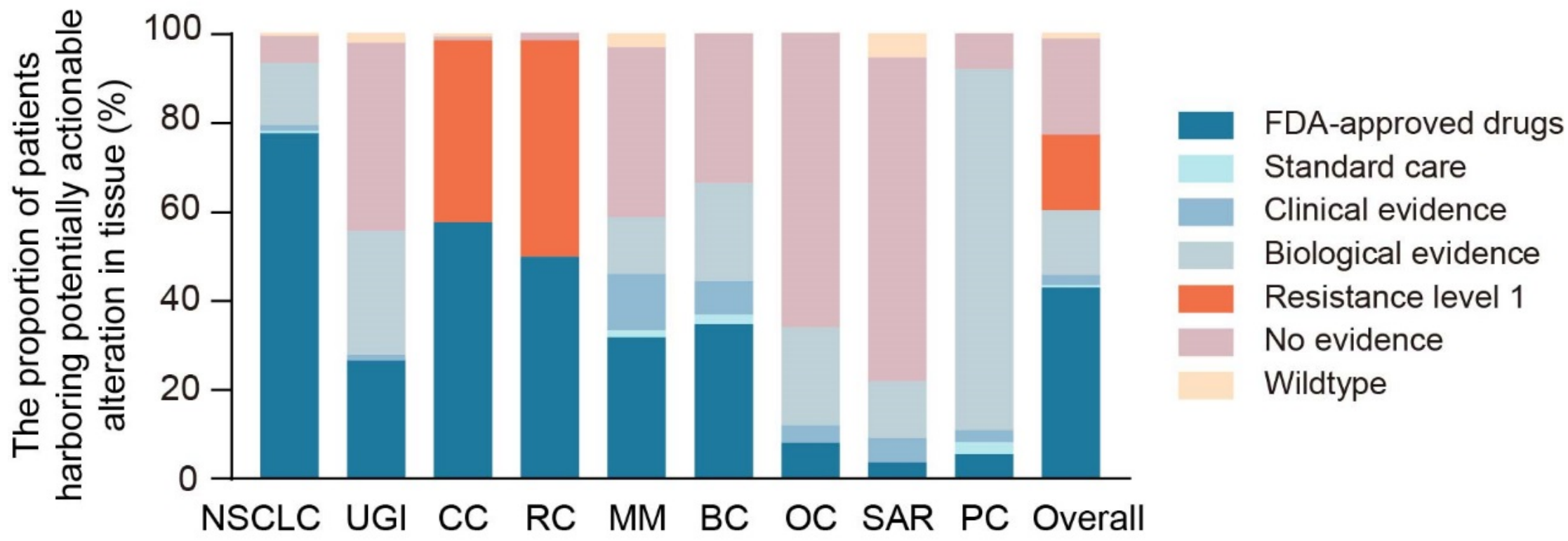


Supplementary Fig. 9 The proportion of patients harboring potentially actionable alteration in tissue. NSCLC, non-small cell lung cancer; UGI, upper gastrointestinal cancer; CC, colon cancer; RC, rectal cancer; MM, melanoma; BC, breast cancer; OC, ovarian cancer; SAR, sarcoma; PC, pancreatic cancer.

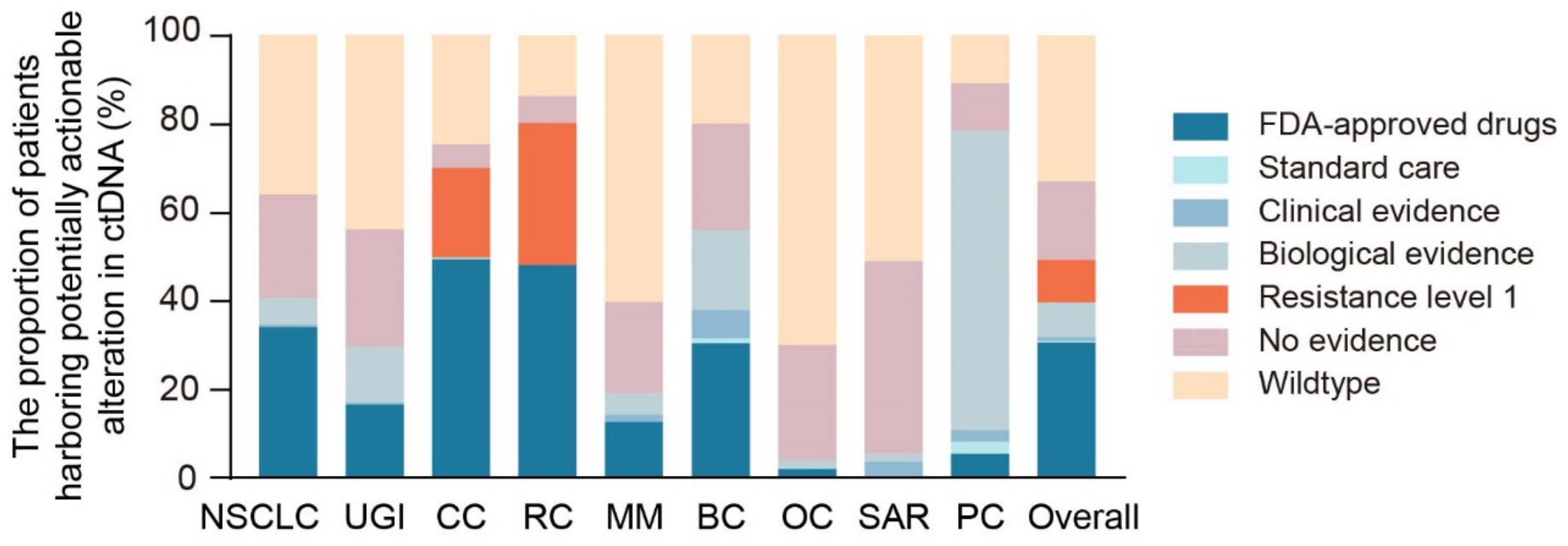


Supplementary Fig. 10 The proportion of patients harboring potentially actionable alteration in ctDNA. NSCLC, non-small cell lung cancer; UGI, upper gastrointestinal cancer; CC, colon cancer; RC, rectal cancer; MM, melanoma; BC, breast cancer; OC, ovarian cancer; SAR, sarcoma; PC, pancreatic cancer.

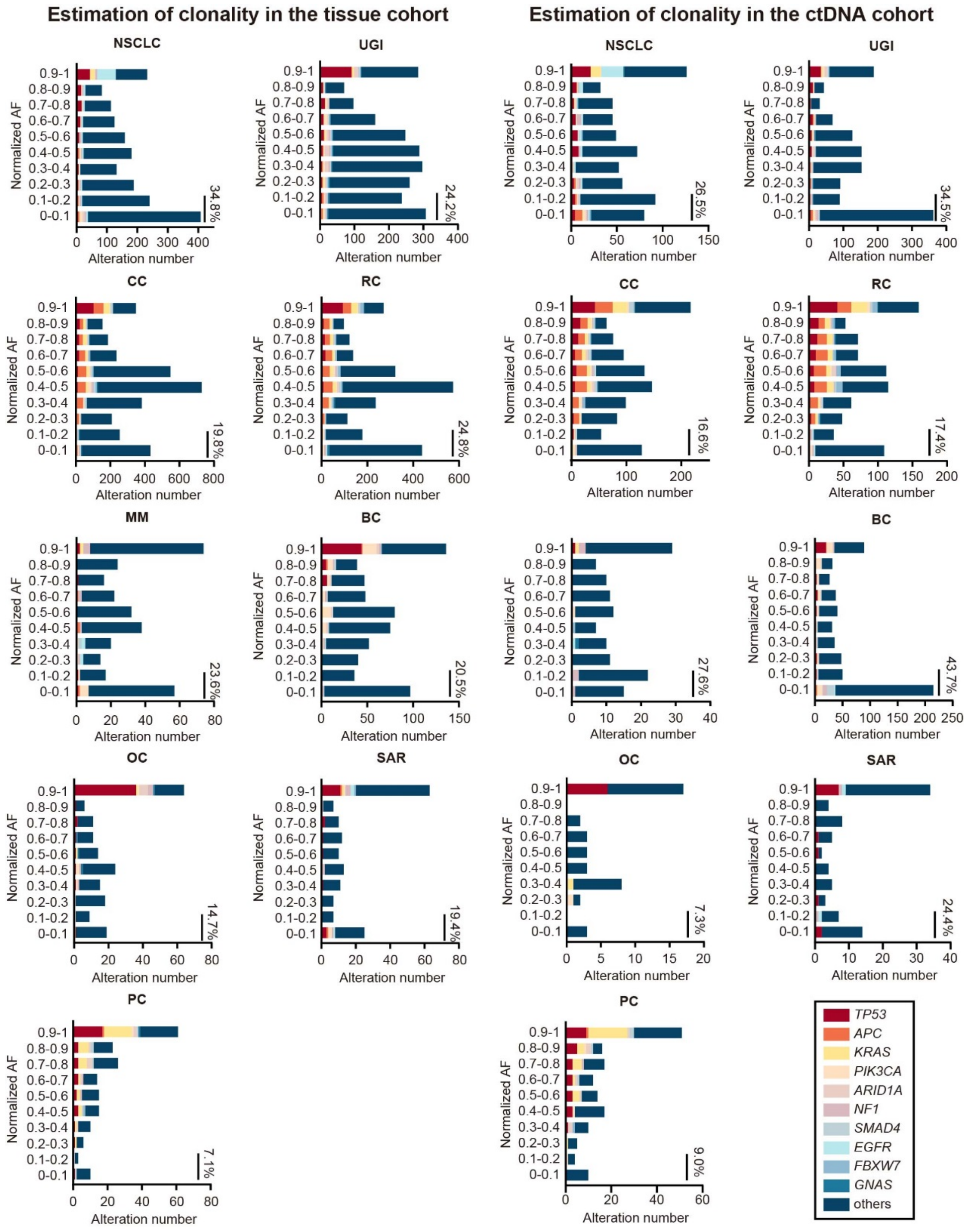


Supplementary Fig. 11 Estimation of clonality among patients with different cancer types in the tissue and ctDNA cohorts. NSCLC, non-small cell lung cancer; UGI, upper gastrointestinal cancer; CC, colon cancer; RC, rectal cancer; MM, melanoma; BC, breast cancer; OC, ovarian cancer; SAR, sarcoma; PC, pancreatic cancer.

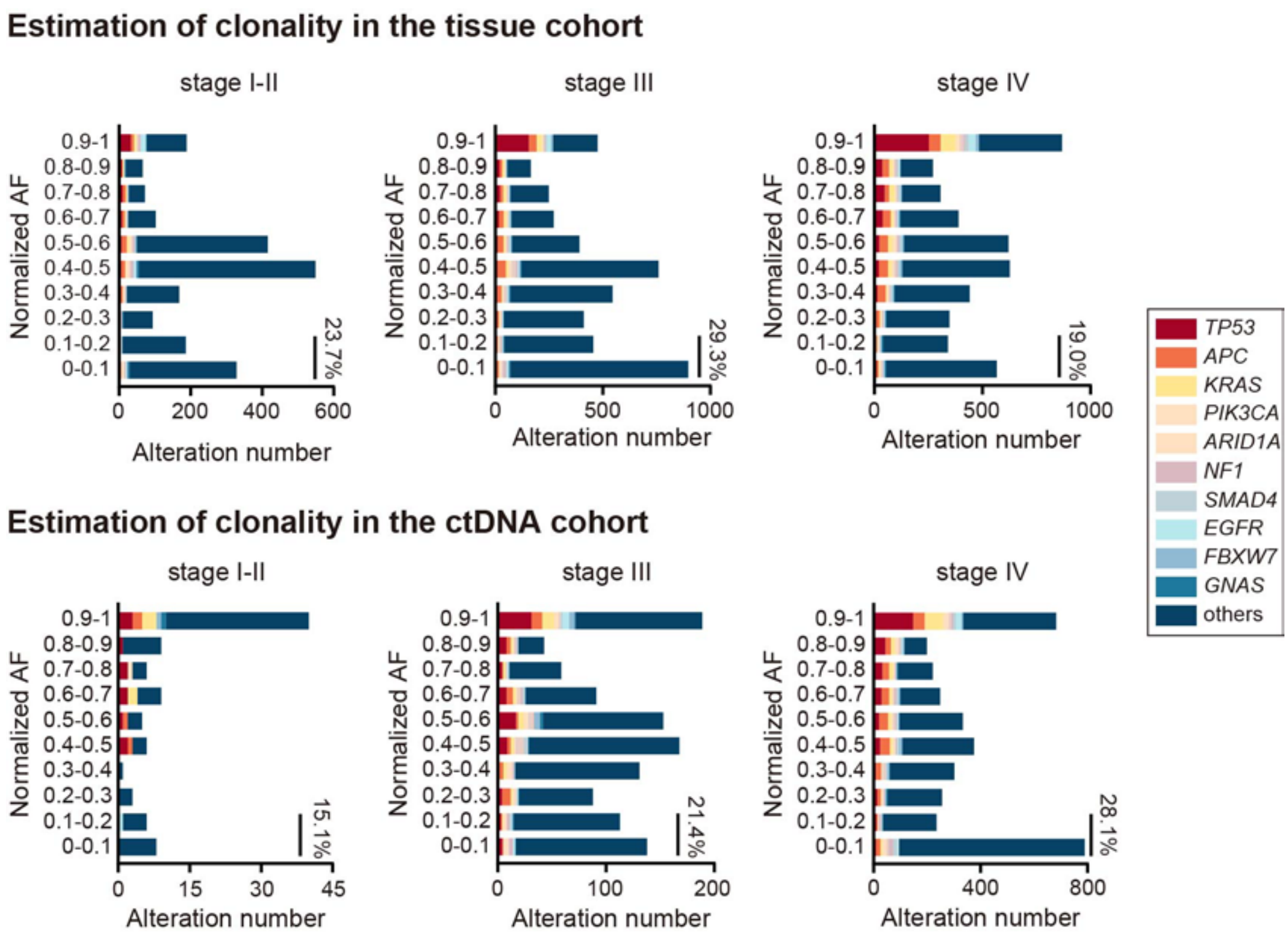


Supplementary Fig. 12 Estimation of clonality among patients with different clinical stages in the tissue and ctDNA cohorts.

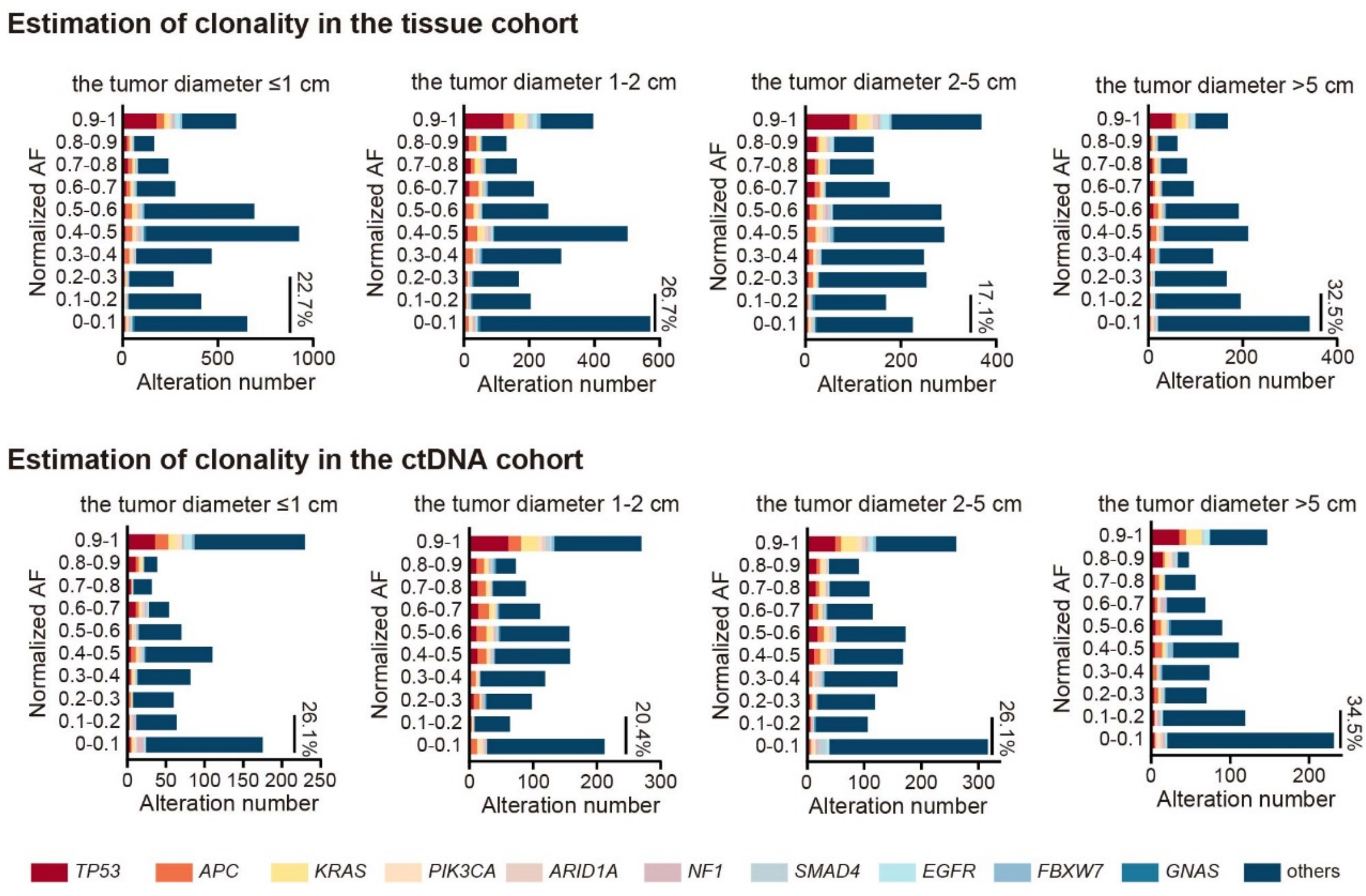


Supplementary Fig. 13 Estimation of clonality among patients with different tumor diameters in the tissue and ctDNA cohorts.

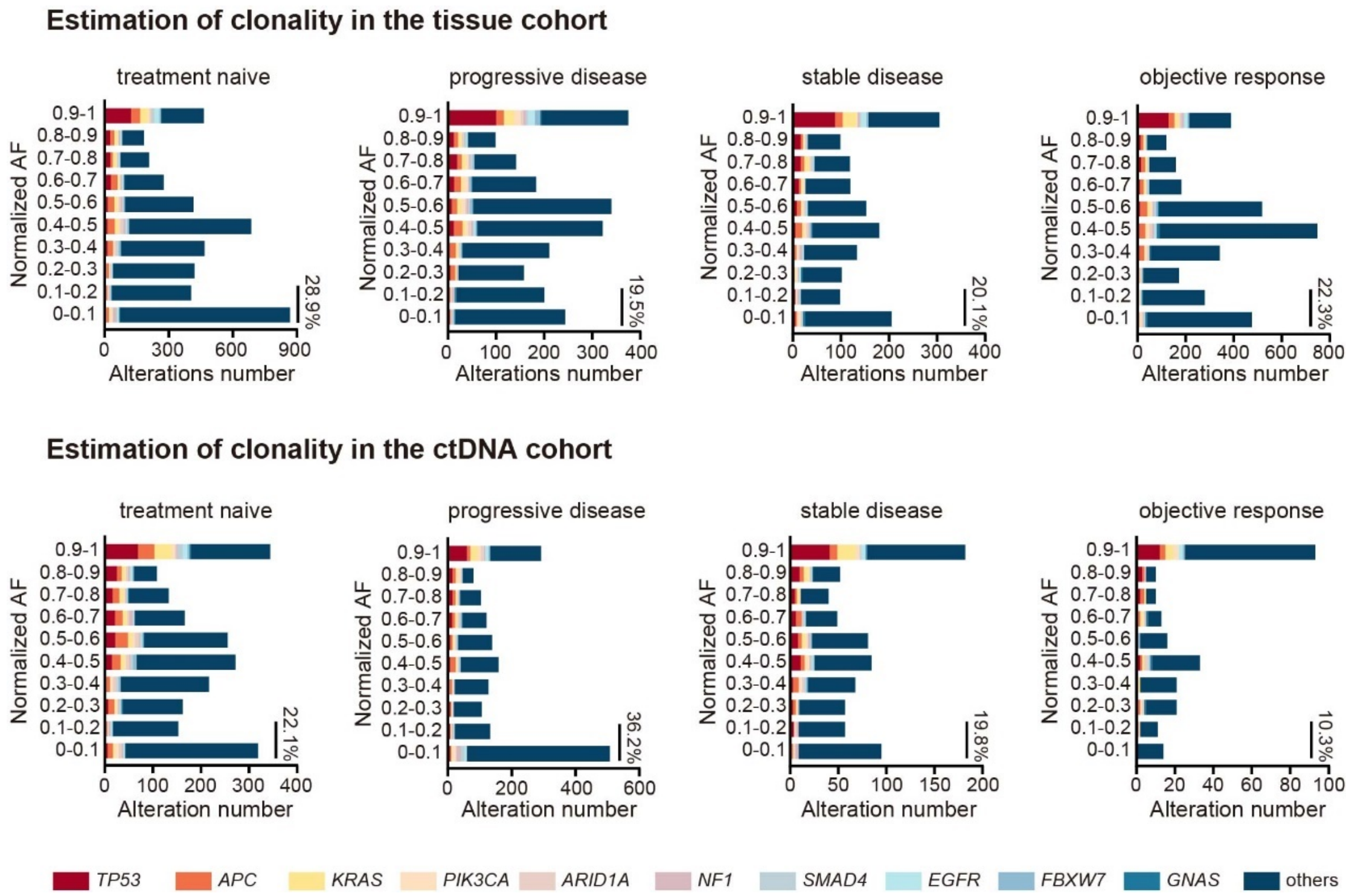


Supplementary Fig. 14 Estimation of clonality among patients with different disease status in the tissue and ctDNA cohorts.

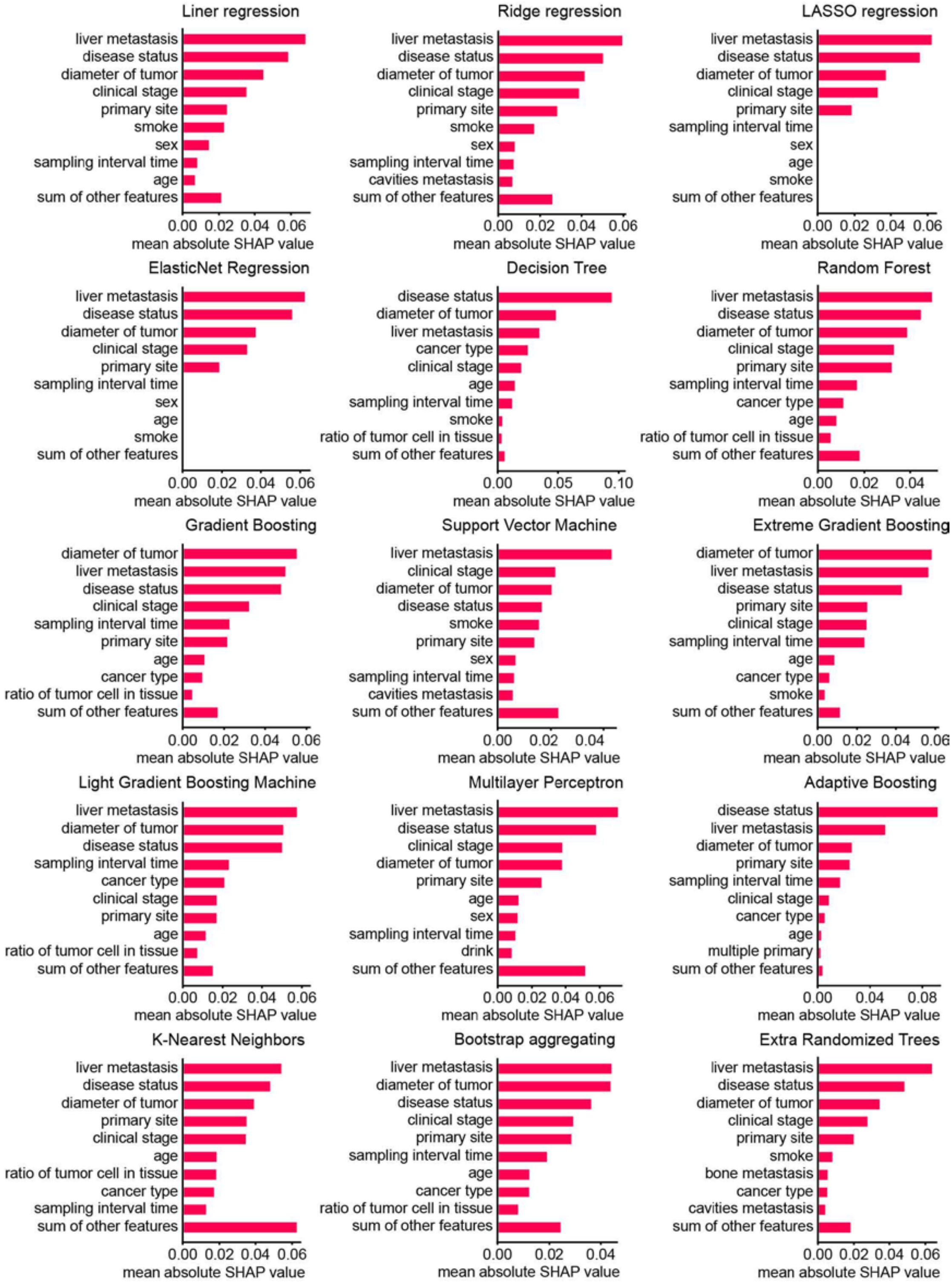


Supplementary Fig. 15 SHAP summary bar plot of 15 machine learning models. The liver metastasis, disease status, and diameter of tumor were the top three importance feature that affected the concordance rate among all models.

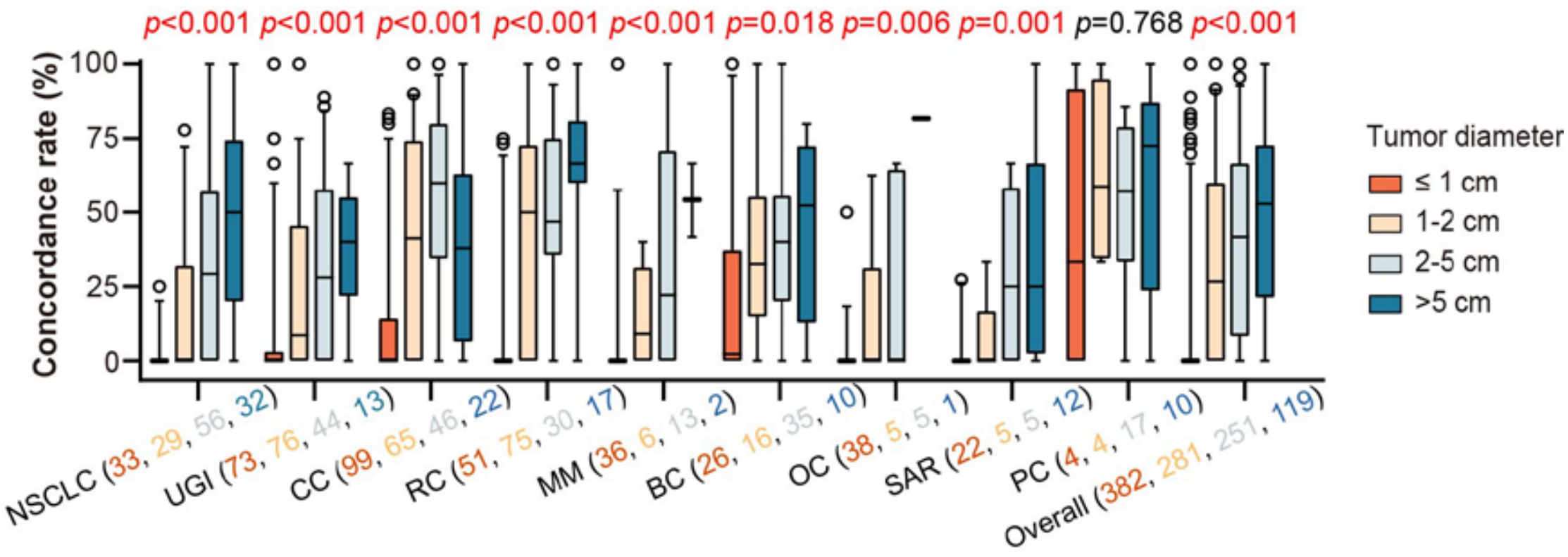


Supplementary Fig. 16 The effect of tumor diameter on the concordance of somatic variations between ctDNA and tissue among different cancer types. NSCLC, non-small cell lung cancer; UGI, upper gastrointestinal cancer; CC, colon cancer; RC, rectal cancer; MM, melanoma; BC, breast cancer; OC, ovarian cancer; SAR, sarcoma; PC, pancreatic cancer.

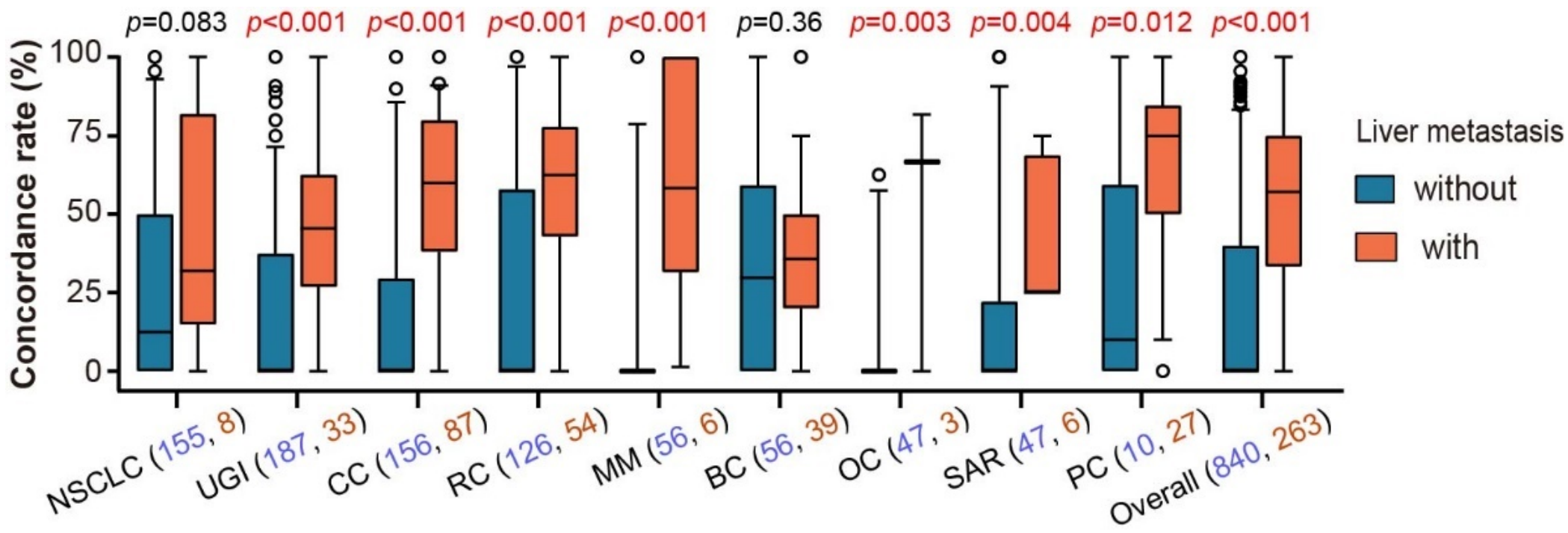


Supplementary Fig. 17 Group comparisons of concordance rate among patients with different liver metastasis statuses in each cancer type and the overall cohort. NSCLC, non-small cell lung cancer; UGI, upper gastrointestinal cancer; CC, colon cancer; RC, rectal cancer; MM, melanoma; BC, breast cancer; OC, ovarian cancer; SAR, sarcoma; PC, pancreatic cancer.

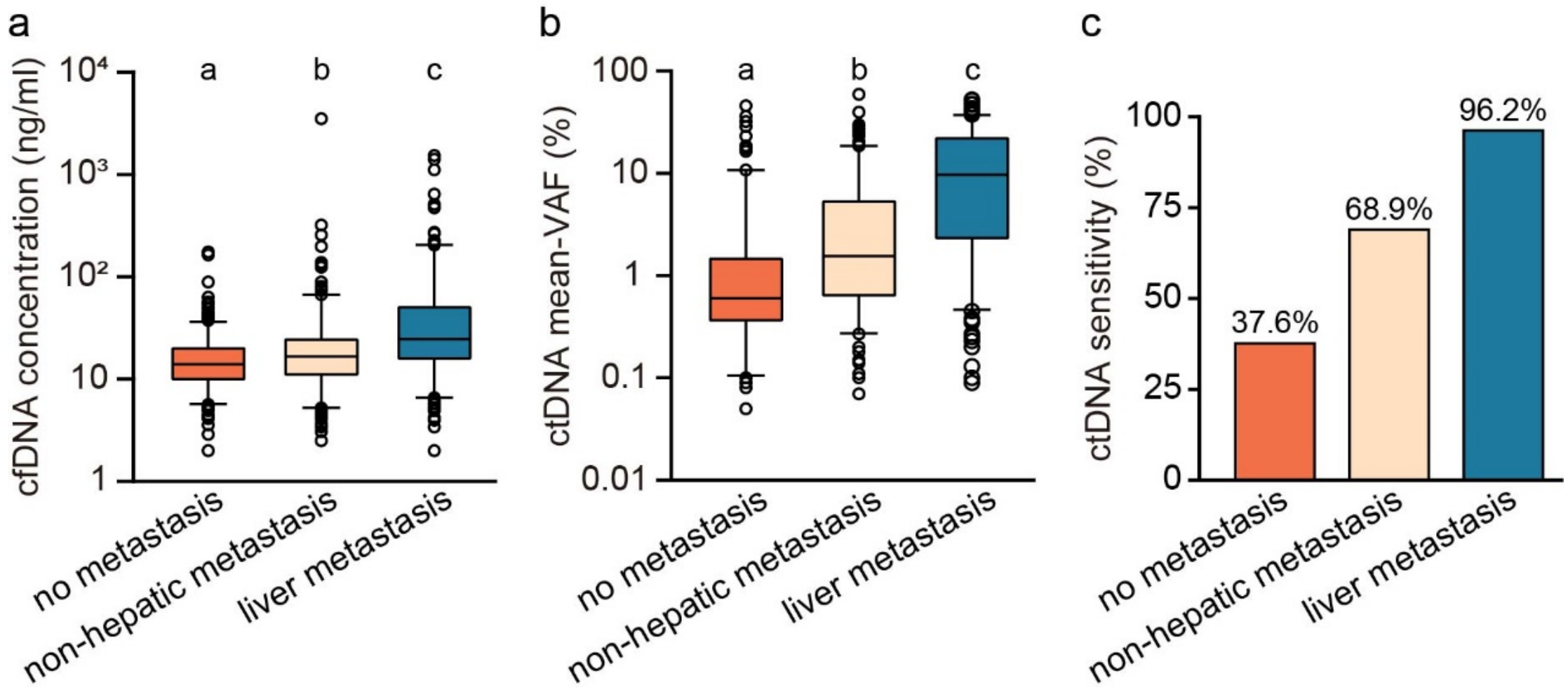


Supplementary Fig. 18 The concentration of cfDNA extracted from plasma, mean variant allele frequency (mean-VAF) of mutations in ctDNA, and detection sensitivity of ctDNA varied across different liver metastasis statuses. Letters above boxplots denote statistically homogeneous subsets determined by post hoc Dunn's testing following Kruskal-Wallis H analysis. Groups sharing at least one common letter indicate no significant pairwise difference.

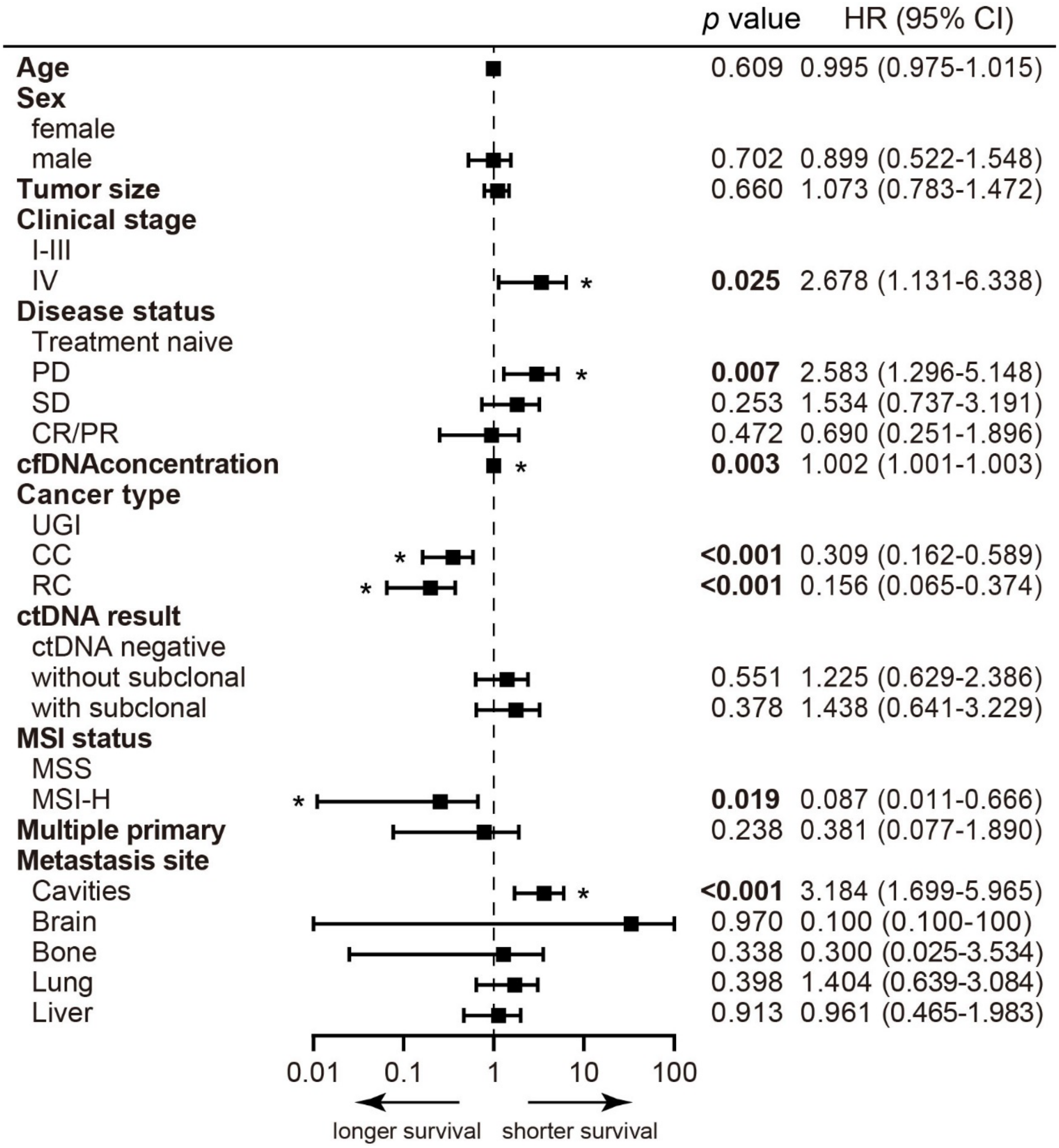


Supplementary Fig. 19 Gastrointestinal-specific multivariate survival analysis
